# Intuitive judgements towards artificial intelligence verdicts of moral transgressions

**DOI:** 10.1111/bjso.12908

**Published:** 2025-05-31

**Authors:** Yuxin Liu, Adam Moore

**Affiliations:** ^1^ School of Philosophy, Psychology and Language Sciences The University of Edinburgh Edinburgh UK; ^2^ Centre for Technomoral Futures, Edinburgh Futures Institute The University of Edinburgh Edinburgh UK

**Keywords:** automated decision‐making, belief alignment, human–AI interaction, moral intuitions, motivated reasoning

## Abstract

Automated decision‐making systems have become increasingly prevalent in morally salient domains of services, introducing ethically significant consequences. In three pre‐registered studies (*N* = 804), we experimentally investigated whether people's judgements of AI decisions are impacted by a belief alignment with the underlying politically salient context of AI deployment over and above any general attitudes towards AI people might hold. Participants read conservative‐ or liberal‐framed vignettes of AI‐detected statistical anomalies as a proxy for potential human prejudice in the contexts of LGBTQ+ rights and environmental protection, and responded to willingness to act on the AI verdicts, trust in AI, and perception of procedural fairness and distributive fairness of AI. Our results reveal that people's willingness to act, and judgements of trust and fairness seem to be constructed as a function of general attitudes of positivity towards AI, the moral intuitive context of AI deployment, pre‐existing politico‐moral beliefs, and a compatibility between the latter two. The implication is that judgements towards AI are shaped by both the belief alignment effect and general AI attitudes, suggesting a level of malleability and context dependency that challenges the potential role of AI serving as an effective mediator in morally complex situations.

## INTRODUCTION

Contrary to the idiom, facts do not speak for themselves. Research in social and political psychology, particularly on identity politics and in‐group/out‐group partisanship (Bartels, 2002; Cohen, 2003), shows identical incoming information can be processed in distinctively different ways, that is individuals tend to accept/reject incoming information and react to the behaviour of others as a function of (mis)alignment with their existing ideology/worldview (Cook & Lewandowsky, 2016; Cram et al., 2018; Drummond & Fischhoff, 2017; Gaines et al., 2007; Hameleers & van der Meer, 2020; Jern et al., 2014; Moore et al., 2021). Notably, this kind of selective distortion can occur regardless of factual accuracy, or even at the expense of it, for a host of political/politicized issues such as climate change (McCright & Dunlap, 2011), immigration and asylum seekers (Glinitzer et al., 2021) and Brexit (Hobolt et al., 2021). Such selective information processing has been more often explained by accounts of motivated reasoning (Flynn et al., 2017; Jost, 2017; Jost et al., 2003, 2017; Jost & Amodio, 2012; Jost & Krochik, 2014; Kahan, 2016a, 2016b; Krochik & Jost, 2011; Moore et al., 2021; Taber & Lodge, 2006) than by accounts of effortful rejection of information (Pennycook & Rand, 2019; Roozenbeek & van der Linden, 2019).

Whilst these self‐perpetuating belief systems and associated ideological/affective polarization (Geschke et al., 2019; Iyengar et al., 2019; van Baar & FeldmanHall, 2021) present inherent challenges, they are further complicated by technological development that raises concerns over fairness/biases, mis/disinformation, opacity/explainability and more (Weidinger et al., 2022). These low‐trust socio‐political environments with entrenched belief polarization, nonetheless, also present opportunities for third‐party agents to provide guidance – could AI effectively fulfil this role? The term ‘AI’ here refers to a broad umbrella of decision‐assisting technologies. For example, earlier studies in human–computer interaction tend to focus on statistical forecasting algorithms and the resulting human preference for, or prejudice against, algorithmic predictions (Dietvorst et al., 2015; Logg et al., 2019). More recently, advancements in large language models have shifted attention towards examining the perception of AI‐generated human‐like utterances and their persuasive impact on users' judgements and beliefs (Costello et al., 2024). In the adjacent field of machine ethics that seeks to embed moral capacity in robots, machines and AI, several theoretical proposals have emerged for building artificial moral advisors using various philosophical frameworks and design approaches (Giubilini & Savulescu, 2018; Lara & Deckers, 2020; but see Liu et al., 2022).

These developments raise a critical question: to what extent do individuals' pre‐existing intuitions and beliefs about a given topic shape their judgements towards AI‐generated output? This is a key concern because, if motivated reasoning drives user evaluation of AI beyond general attitudes towards AI, then the potential for AI systems to serve as mediators in morally/politically contentious situations will be significantly undermined. More concerning is the risk of asymmetric acceptance of AI‐generated content, where individuals dismiss AI as biased/unreliable when it challenges their pre‐existing beliefs but accept it as independent/trustworthy when it aligns with them. Given the growing prevalence of AI operating in morally salient contexts, it is crucial to understand how people assess AI as a mediator in complex moral decision‐making processes.

Extensive research has been conducted on human–AI/computer interaction, and more recently, on the moral psychology of AI (Bonnefon et al., 2024; Ladak et al., 2023). Earlier studies comparing humans and statistical algorithms have often demonstrated an algorithm aversion effect – prejudicial discounting of algorithmic decisions or a preference for human decisions (Dietvorst et al., 2015; Jauernig et al., 2022; Longoni et al., 2019; Morewedge, 2022; Önkal et al., 2009; Prahl & van Swol, 2017; Zhang et al., 2021), though fewer studies show the opposite – an algorithm appreciation (Logg et al., 2019; see Burton et al., 2020; Mahmud et al., 2022 for systematic reviews). Beyond preferences, abundant research has explored moral norms, fairness, responsibility and accountability in judgement and decision‐making involving AI (Araujo et al., 2020; Banks, 2020; Bonnefon et al., 2016; Hong et al., 2020; Kahn et al., 2012; Malle et al., 2015, 2019; Shank et al., 2019, 2021; Shank & DeSanti, 2018; Shariff et al., 2017), though definitive conclusions remain elusive.

These studies tend to focus on elements related to the internal design of algorithms, individual differences in psychological features, task characteristics and higher‐level (e.g. cultural/societal) factors (Mahmud et al., 2022). Task characteristics, for example, are crucial in AI perception: individuals display greater trust in, and perceived fairness of, AI, for tasks perceived as objective/quantifiable, as opposed to subjective/associated with human affective abilities (Castelo et al., 2019; Lee, 2018). Though many studies considered the influence of familiarity or experience with AI, the role of general attitudes towards AI is often overlooked, and more importantly, so is whether the AI output agrees with participants' pre‐existing socio‐moral beliefs. The potential effects of motivated reasoning in human–AI interactions are particularly relevant because, unlike static features of the technology/individual user, pre‐existing beliefs and ideologies can be amplified through repeated interactions with AI systems and diffused across social networks, reinforcing and spreading biased judgements over time (Glickman & Sharot, 2025).

### The current research

Given the tendency to evaluate incoming information based on its compatibility with existing beliefs, does this pattern extend to information coming from an AI? And if so, does this pattern hold beyond any intuitions that people might have about AI itself? Using vignettes of narrow AI deployment, we aim to investigate whether judgements towards AI are impacted by belief alignment with the AI's output in its deployment context over and above general AI attitudes. Though we do control for general/broader AI attitudes, focusing on a narrow AI with a specific, pattern‐detection function allows us to target this (mis)alignment somewhat separately from potential assumptions about more complex, human‐like AI.

To reliably elicit polarized beliefs independent of general AI attitudes, these scenarios involve politically salient contexts (LGBTQ+ rights and environmental protection), which allow us to examine whether AI detection of potential bias in human behaviour is considered sufficient evidence for transgressions that warrant investigative actions. We experimentally manipulate the (mis)alignment between AI recommendation and participants' pre‐existing politico‐moral intuitions for the underlying context, aiming to prompt a compatibility or conflict between them. The rationale is that if judgements towards AI output are contingent on pre‐existing intuitions related to the context, then belief alignment should lead to a greater acceptance of AI verdicts, and vice versa, notwithstanding any general intuitions people might have about AI.

Additional to willingness to act, judgements of trust and perception of fairness are also two prevalent dimensions of moral judgements and are both linked to reactions to decision outcomes (Bianchi et al., 2015; Skitka & Mullen, 2002; Tyler & Degoey, 1996; Tyler & Smith, 1999). They are not only frequently studied in judgements towards different types of authorities/experts (de Cremer & Tyler, 2007; Promberger & Baron, 2006), but also in human–machine interactions (Castelo et al., 2019; Grgić‐Hlača et al., 2018, 2022; Lee, 2018; Lee & Baykal, 2017; Lee & Rich, 2021; Malle & Ullman, 2021; Starke et al., 2022; Ullman & Malle, 2018, 2019). The majority of literature on fairness perceptions in algorithmic decisions (Starke et al., 2022) focuses on identifying algorithmic or human predictors of fairness perception of AI and testing the consequences of perceived fairness. Relatively more research has investigated trust(worthiness) of AI in a similar fashion (see Bach et al., 2022; Ueno et al., 2022 for systematic reviews). This includes examining technical design features and human factors that enhance or undermine trust in human–machine interaction, devising measurement of trust in machines (Malle & Ullman, 2021; Ullman & Malle, 2018, 2019), comparing trust in human/AI (Liang & Newell, 2022) and exploring relationships between trust and other variables (Choung et al., 2023). As such, we will also investigate whether trust and fairness perception are subject to context‐based motivated reasoning in cases of AI/automated decisions.

Lastly, considerable research in political psychology reveals an ideological asymmetry, such that conservatives, compared to liberals, tend to score higher on variables such as cognitive rigidity and dogmatism, and lower on integrative complexity, cognitive reflection, and non‐political measures of cognitive flexibility (Amodio et al., 2007; Jost, 2017; Zmigrod et al., 2020). Thus, albeit not the main objective, we expect to observe a greater belief (mis)alignment effect for conservatives, given that on average, conservatives are more susceptible to various cognitive and epistemic biases (Baron & Jost, 2019) and political conservatism may be, in part, a manifestation of motivated social cognition (Jost et al., 2003).

We conducted three pre‐registered experiments (https://osf.io/7qjt3). In E1 (https://osf.io/wufpg) and E2 (https://osf.io/q82ec), we examined how willingness to act on AI verdicts of moral transgression, trust in AI and perceived fairness of AI varied with alignment between participants' overall political orientation and AI recommendations beyond general AI attitudes in contexts of LGBTQ+ rights and environmental concerns. In E3 (https://osf.io/wm29n), we used issue‐specific measures to better capture the alignment in those contexts. Results consistently showed that belief alignment impacted judgements towards AI verdicts over and above general AI attitudes, suggesting a level of malleability and context dependency that challenges AI's potential mediating role in morally charged situations.

## EXPERIMENT 1 AND 2

Given the well‐documented tendency for motivated reasoning, judgements about AI outcomes may be predominantly driven by context‐based belief alignment depending on the compatibility between AI verdicts of moral transgressions and pre‐existing politico‐moral beliefs. We predict: (1) increased willingness to act on AI recommendations, increased trust in AI and increased perception of fairness of AI when those AI recommendations align with participants' pre‐existing moral intuitions, compared to when they do not align (i.e. a belief alignment effect), (2) belief alignment will occur over and above general AI attitudes and (3) conservatives showing stronger belief alignment than liberals.

We conducted two experiments using the same study design and materials: E1 was a repeated‐measures design (multiple scenarios per participant) and E2 was between‐subjects (one scenario per participant). A consistent pattern of results across the two experiments should increase our confidence in the effect of belief alignment.

### METHODS

#### Participants

After excluding one participant from each experiment for failing the attention check, we had 201 (67 males and 131 females; *M*_age_ = 36.70 years, *SD*_age_ = 13.38 years) and 301 native English‐speaking adult participants (109 males and 190 females; *M*_age_ = 37.68 years, *SD*_age_ = 14.11 years) in E1 and E2, respectively. Testing was conducted online via Qualtrics integrated into the crowdsourcing platform Prolific to recruit diverse, representative, attentive and naïve subjects (Palan & Schitter, 2018; Peer et al., 2017, 2021). Participants were compensated £0.84 for E1 and £0.59 for E2, and repeat participation was prevented via Prolific internal filtering.

#### Design and materials

We collected data on (1) basic demographics and political orientation, (2) general attitudes towards AI and (3) intuitive responses to hypothetical scenarios of AI verdicts described as AI‐detected statistical anomalies indicative of moral transgressions. These three sections were presented in a random order.

##### Demographic information

We collected participants' age, gender and aspects of political orientation. To account for different underlying political attitudes associated with facets of conservatism (Crowson, 2009; Harnish et al., 2018; Pratto et al., 1994), we measured political positions via one question each on economic, social and foreign policy views (‘Using the following scale, how left‐wing/liberal or right‐wing/conservative are you on economic/social/foreign policy issues?’; 1 = *very left‐wing/liberal* to 7 = *very right‐wing/conservative*).

##### General attitudes towards AI

We used the General Attitudes towards Artificial Intelligence Scale (GAAIS; Schepman & Rodway, 2020, 2022) to measure overall sentiments towards AI in the general public. The GAAIS consists of a positive subscale – 12 items capturing the practical functionality and potential societal/personal benefits of AI applications (e.g. ‘I am interested in using artificially intelligent systems in my daily life’; 1 = *strongly disagree* and 5 = *strongly agree*), and a negative subscale – eight items capturing dystopian concerns towards the presumed danger of AI (e.g. ‘I think artificial intelligence is dangerous’; 1 = *strongly agree* and 5 = *strongly disagree*). Negative items were reverse‐coded so that higher ratings on both subscales would indicate more positive general attitudes towards AI. We calculated subscale means separately as instructed, due to the lack of unidimensionality.

##### Hypothetical scenarios

We created eight scenarios where organizations use a reliable expert AI system to evaluate their everyday operations, and the AI system detects statistically anomalous decisions made by a human agent in the organization (e.g. Table 1). We described a statistical pattern‐detection algorithm here, rather than a more topical generative AI, for simplicity and to minimize potential confounding variables such as perceptions of agency, autonomy and humanness. Statistical anomalies as an insinuation of potential bias allow for a more focused study design without implicit assumptions of more sophisticated AI systems. We return to this point in the General Discussion.

**TABLE 1:** Example hypothetical scenarios.

Left‐wing/Liberal context	Right‐wing/Conservative context
A *banking oversight committee* has been using an efficient and reliable artificial intelligence system called Analytic Intellect to analyse loan application outcome patterns. The AI detected that a particular loan manager has been anomalously more likely to *reject* mortgage loan requests submitted by *same‐sex couples.*	A leading technology company has partnered with the Ministry of Justice to develop and train an artificial intelligence named LEA (Legal Expert Assistant) to serve *judicial needs*. The main objective of this AI is to identify any statistical anomalies in civil judicial decisions, which would potentially be flagged for re‐evaluation. When reviewing the results of environmental claims cases in the past year, LEA detected that a particular judge has been ruling *in favour of* claims against corporations in *pollution or environmental damage* cases at a significantly higher rate than average.

*Note*: Italics indicate domains, actions and foci for clarity; no text was italicized for the participants. Overall length of scenarios did not differ as a function of context alignment.

These scenarios represent a fully factorial 2 (Context: Left‐wing/Liberal or Right‐wing/Conservative moral intuitive direction) × 2 (Approve or Reject action taken by the human agent) × 2 (Financial or Judicial domain of the scenario) design. Context indicates an AI verdict compatible with either liberal or conservative moral intuitions (e.g. an AI flagging a judge for prejudice against same‐sex couples aligns with left‐wing/liberal intuitions that such discrimination is wrong and should be stopped). Approve/reject action indicates the human agent favouring or discriminating against a target. Domain indicates the superficial setting of the scenarios (financial bank or judicial court), which are nested in a person‐centred (LGBTQ+ rights) and a cause‐centred (environmental concerns) focus. All elements (context, action, domain and focus) are counterbalanced.

Participants responded to three statements following each scenario, with each statement presented on a continuous slider (1 = *strongly disagree* to 5 = *strongly agree*) with a midpoint default. Willingness to act on AI recommendations refers to participants' support for default interventions (e.g. an investigation) based solely on AI detection of possible prejudice (‘Based on the AI's recommendation, I think that this person in the scenario should be investigated’). Trust in AI refers to the extent to which participants perceive the AI judgement as trustworthy (‘I trust the AI's judgement in this case’). Perceived fairness of AI refers to the extent to which they perceive the AI as fair and appropriate (‘I believe that the AI is being fair in this case’).

#### Procedures

After giving informed consent, participants completed the above‐mentioned three sections in a random order. E1 participants received two pseudo‐randomly selected scenarios from opposite factorial cells in each topical focus, and E2 participants were shown one randomly selected scenario out of the eight possibilities. After each scenario, participants responded to statements on willingness to act, trust and perceived fairness, one at a time on separate pages while the given scenario remained visible above each statement. For both experiments, all scenarios were approximately evenly presented across participants.

#### Statistical analysis plan

We opted for Bayesian statistical analysis (using *brms* package (v. 2.19.0); Bürkner, 2017, 2018) in RStudio (v. 4.3.0; R Core Team, 2023) to quantify support for our hypotheses of interest, rather than the (in)compatibility of the evidence with the null hypothesis (McElreath, 2015). Under the Bayesian framework, we computed zero‐order correlations and multilevel multivariate multiple regression models, with the main pre‐registered model containing the predictors of interest, covariates and a nuisance variable (see below).

All continuous variables were standardized prior to analysis. We averaged the three standardized political view items to obtain an overall measure of participant political position, where higher scores indicate increasing right‐wing conservatism. Fixed effects of context, participant political orientation and the interaction of the two were entered into the models as main predictors of interest. We used a prior of *β*~normal (0, .25) for the intercept, and weakly regularizing priors of *β*~normal (0, 1) for context and political orientation, whose effects were not explicitly predicted. For the interaction term between those two, the prior was set at *β*~normal (.3, .15), indicating the hypothesized increases in willingness to act, trust and fairness perception as a function of belief alignment. Means of positive and (reverse‐coded) negative subscales of GAAIS were entered as covariates of interest to account for participants' general views of AI unrelated to our scenario design. Both GAAIS subscales had a prior of *β*~normal (.2, .1) to indicate our expectation that general optimism towards AI would likely predict more positive judgements about AI verdicts, but to a lesser degree compared to belief alignment effect. Age was also included as a nuisance covariate to represent basic familiarity with AI, with a prior of *β*~normal (−.1, .1) to account for its small negative effect on perception of technologies (Schepman & Rodway, 2020, 2022). Unique idiosyncrasies within each item, topic and individual subject were modelled with random intercepts. All the above parameters and priors were used to simultaneously predict willingness to act on AI verdicts, trust in AI and perceived fairness of AI, thus controlling for correlations amongst them and generating unique predictive effects for each outcome.

Posterior distributions of regression parameters were derived by simulation using Markov chain Monte Carlo (MCMC) estimation (Betancourt, 2018; Bürkner, 2017, 2018; Gelman & Rubin, 1992). For all models, we sampled from four independent MCMC chains with 1000 burn‐in samples and 15,000 sampling iterations per chain. All models converged (all $\hat{R}$ *s* = 1.0; Brooks & Gelman, 1998; Gelman et al., 2013; Gelman & Rubin, 1992). Effect size uncertainty is computed as 95% highest density intervals (HDIs) around the posterior mean, where θ ∈ 95% HDI would indicate a 95% credibility that the true parameter value lies within this range (Kruschke, 2014; McElreath, 2015).

### Results

#### Descriptive statistics and correlations

Descriptive statistics (Table 2) show consistent distributions across E1 and E2. Both participant samples were left‐leaning, with 60.7% (E1) and 57.5% (E2) scoring an average political position below four, as opposed to 20.4% (E1) and 19.3% (E2) above four on the seven‐point Likert scale, where higher scores indicate greater conservatism. Replicating Schepman and Rodway's (2020, 2022) results with good internal consistency, participants in both samples generally held positive attitudes towards beneficial utilities of AI (*α*_E1_ = 0.89, *α*_E2_ = 0.88) and lower dystopian concern (*α*_E1_ = 0.83, *α*_E2_ = 0.84). Participants also showed a moderate willingness to accept and act on the statistical AI verdicts of potential prejudice, placed trust in the AI system to detect such anomalies and perceived the AI judgements as fair.

**TABLE 2:** Descriptive summaries of measured variables in Experiment 1 and 2.

	Experiment 1 (within‐subjects)			Experiment 2 (between‐subjects)		
	Mean (*SD*)	Median	Range	Mean (*SD*)	Median	Range
Political positions (1 = *Very Left/Liberal*, 7 = *Very Right/Conservative*)						
Economic issues	3.39 (1.33)	3	6	3.47 (1.34)	4	6
Social issues	3.14 (1.39)	3	6	3.16 (1.32)	3	6
Foreign policy issues	3.37 (1.34)	4	6	3.40 (1.40)	4	6
Mean political position	3.30 (1.25)	3.33	5.67	3.34 (1.25)	3.33	6
General attitudes towards AI (5‐point Likert scale; higher score indicates positive attitudes)						
Positive subscale	3.33 (0.60)	3.33	2.75	3.31 (0.60)	3.33	3.5
Negative subscale	2.97 (0.65)	3	3.25	3.04 (0.69)	3.12	3.75
Responses to scenarios (1 = *Strongly Disagree*, 5 = *Strongly Agree*)						
Willingness to act	3.94 (0.91)	4.07	4	3.90 (0.93)	4.07	4
Trust	3.56 (0.86)	3.63	4	3.44 (0.92)	3.7	4
Perceived fairness	3.68 (0.92)	3.95	4	3.56 (0.94)	3.78	4

*Note*: For meaningful interpretations, descriptive statistics are presented in original scales.

Bayesian Pearson's zero‐order correlations for E1 and E2 are displayed in Table 3. In E2 only, participant political position was negatively related to both subscales of general AI attitudes. The latter were correlated with each other in both E1 and E2, as higher scores on both positive and (reverse‐coded) negative GAAIS subscales indicated more positive attitude towards AI. In E1 only, both GAAIS subscales weakly correlated only with trust in AI and perceived fairness of AI. Notably, in both experiments, trust and perceived fairness were more strongly correlated to each other than either was to willingness to act.

**TABLE 3:** Bayesian Pearson's zero‐order correlations and their 95% HDIs between main variables in Experiment 1 (E1; lower diagonal) and Experiment 2 (E2; upper diagonal).

E1	E2					
	Political positions	GAAIS positive	GAAIS negative	Willingness to act	Trust	Perceived fairness
Political positions	1	−0.13** [−0.24, −0.02]	−0.13** [−0.25, −0.02]	0.03 [−0.09, 0.14]	0 [−0.1, 0.12]	−0.06 [−0.17, 0.06]
GAAIS positive	−0.07 [−0.16, 0.03]	1	0.5*** [0.41, 0.58]	0.07 [−0.04, 0.18]	−0.01 [−0.12, 0.11]	0.02 [−0.09, 0.13]
GAAIS negative	0.05 [−0.04, 0.14]	0.51*** [0.43, 0.57]	1	0 [−0.11, 0.11]	−0.02 [−0.13, 0.09]	0.02 [−0.09, 0.14]
Willingness to act	0 [−0.1, 0.1]	0.07 [−0.03, 0.17]	0.06 [−0.03, 0.16]	1	0.35*** [0.26, 0.45]	0.36*** [0.26, 0.46]
Trust	−0.02 [−0.12, 0.07]	0.19*** [0.1, 0.29]	0.15*** [0.05, 0.24]	0.3*** [0.21, 0.39]	1	0.63*** [0.56, 0.69]
Perceived fairness	−0.06 [−0.16, 0.03]	0.21*** [0.11, 0.3]	0.11* [0.01, 0.2]	0.36*** [0.27, 0.44]	0.61*** [0.55, 0.67]	1

*Note*: Probability of direction (pd) represents the portion of the posterior distribution in the same direction of effect as the median (Makowski et al., 2019). GAAIS negative values are reverse‐coded.

* pd. > 97.5%.

** pd. > 99.5%.

*** pd. > 99.95%.

#### Bayesian regression analysis

**FIGURE 1:**
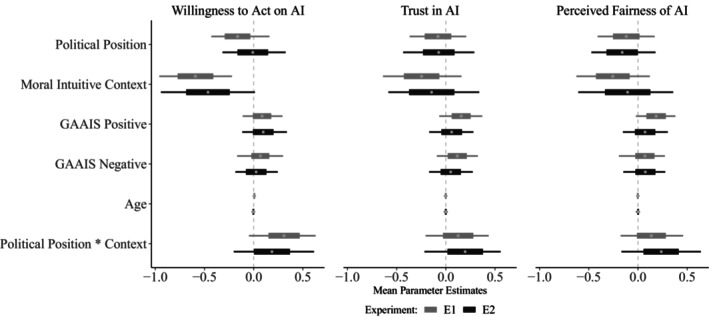
Parameter estimates for Willingness to Act on AI verdicts, Trust in AI and Perceived Fairness of AI in E1 & E2, with boxes indicating 95% HDIs and whiskers indicating 100% HDIs. Higher standardized scores on political position correspond to increasing conservatism. Context is a binary variable with liberal/left‐wing direction as the reference level.

Our pre‐registered model simultaneously predicted ratings on all three outcome variables (Table 4 & Figure 1). Ratings on willingness to act were negatively predicted by increasing participant political conservatism in E1 (*β* = −.16, 95% HDI = [−0.29, −0.03]) and by the conservative moral intuitive context of AI verdicts in both E1 (*β* = −.59, 95% HDI = [−0.77, −0.41]) and E2 (*β* = −.46, 95% HDI = [−0.69, −0.24]), suggesting that conservatism of both participants and the context was related to less willingness to act on AI verdicts of potential transgression. In E1, but not E2, the conservative context also predicted less trust in AI (*β* = −.25, 95% HDI = [−0.43, −0.07]) and less perceived fairness of AI (*β* = −.26, 95% HDI = [−0.43, −0.08]). Additionally, in E1, those who viewed AI in a positive light were more likely to trust and judge the AI as being fair: both positive (*β* = .16, 95% HDI = [0.06, 0.25]) and reversed negative general AI attitudes (*β* = .12, 95% HDI = [0.02, 0.21]) predicted more trust in AI, and positive general AI attitudes predicted greater perceived fairness of AI (*β* = .18, 95% HDI = [0.09, 0.28]).

**TABLE 4:** Summaries of Bayesian regression results in Experiment 1 and 2.

	Willingness to act		Trust		Fairness perception	
	Mean [95% HDI]	*SD*	Mean [95% HDI]	*SD*	Mean [95% HDI]	*SD*
Experiment 1						
Intercept	0.06 [−0.44, 0.56]	0.25	0.16 [−0.31, 0.63]	0.24	0.18 [−0.29, 0.65]	0.24
Political position	**−0.16 [−0.29, −0.03]**	0.07	−0.08 [−0.21, 0.05]	0.07	−0.12 [−0.25, 0.02]	0.07
Context	**−0.59 [−0.77, −0.41]**	0.09	**−0.25 [−0.43, −0.07]**	0.09	**−0.26 [−0.43, −0.08]**	0.09
GAAIS positive	0.08 [−0.01, 0.18]	0.05	**0.16 [0.06, 0.25]**	0.05	**0.18 [0.09, 0.28]**	0.05
GAAIS negative	0.07 [−0.02, 0.16]	0.05	**0.12 [0.02, 0.21]**	0.05	0.07 [−0.03, 0.17]	0.05
Age	0.01 [0, 0.01]	0	0 [−0.01, 0.01]	0	0 [−0.01, 0.01]	0
Political position × Context	**0.31 [0.15, 0.47]**	0.08	0.13 [−0.03, 0.28]	0.08	0.14 [−0.01, 0.28]	0.07
Experiment 2						
Intercept	0.35 [−0.19, 0.89]	0.27	0.08 [−0.44, 0.59]	0.26	0 [−0.51, 0.51]	0.26
Political position	−0.01 [−0.16, 0.15]	0.08	−0.07 [−0.23, 0.09]	0.08	−0.16 [−0.32, 0]	0.08
Context	**−0.46 [−0.69, −0.24]**	0.11	−0.14 [−0.37, 0.09]	0.12	−0.11 [−0.33, 0.13]	0.12
GAAIS positive	0.1 [−0.01, 0.2]	0.05	0.06 [−0.04, 0.16]	0.05	0.07 [−0.03, 0.18]	0.05
GAAIS negative	0.02 [−0.08, 0.13]	0.05	0.05 [−0.05, 0.15]	0.05	0.08 [−0.03, 0.18]	0.05
Age	0 [−0.01, 0.01]	0	0 [−0.01, 0.01]	0	0 [−0.01, 0.01]	0
Political position × Context	**0.19 [0, 0.37]**	0.09	**0.2 [0.02, 0.38]**	0.09	**0.24 [0.06, 0.41]**	0.09

*Note*: Model converged successfully with split $\hat{R}$ = 1 for all estimated parameters. Context is a binary variable with liberal/left‐wing direction as the reference level. GAAIS Negative values are reverse‐coded. Bold emphasizes 0 ∉ 95% HDI.

**FIGURE 2:**
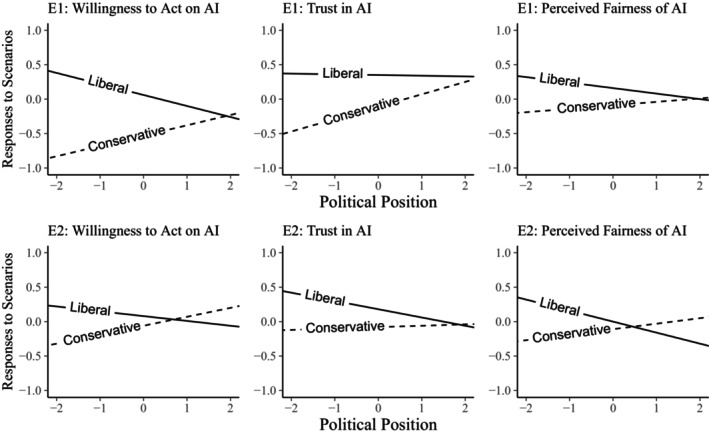
Interactions between participant political position and moral intuitive context of AI verdicts for Willingness to Act on AI verdicts, Trust in AI and Perceived Fairness of AI in E1 & E2. Higher scores on political position correspond to increasing conservatism. Solid and dotted lines indicate liberal and conservative contexts of AI deployment, respectively.

We found a belief alignment effect, indexed by the interaction between participant political conservatism and the conservative moral intuitive context of AI verdicts, for all three responses: willingness to act (*β* = .19, 95% HDI = [0, 0.37]), trust (*β* = .2, 95% HDI = [0.02, 0.38]) and perceived fairness of AI (*β* = .24, 95% HDI = [0.06, 0.41]) in E2, though this was evident only for willingness to act (*β* = .31, 95% HDI = [0.15, 0.47]) in E1. However, these effects were in the opposite direction of our prediction (Figure 2) – left‐wing/liberals showed a stronger increase in Willingness to Act, Trust and Perceived Fairness when the scenario matched their political beliefs, compared to right‐wing/conservatives.

We explored the possibility that belief alignment would increase as positive AI attitudes rose, but model comparisons (LOO‐IC, ELPD, Bayes factor and Bayesian *r* ^2^) indicated such effects did not consistently improve predictive power and strongly favoured the simpler pre‐registered models (see Appendix E.4).

### Discussion

Two main findings emerge from these experiments. First, participants' judgements of trust, fairness perception, and in particular, willingness to act on AI verdicts of moral transgression were lower in contexts that matched conservative moral intuitions, compared to liberal ones. This may be explained by the conservative contexts violating the politico‐moral beliefs of our left‐leaning sample of participants. Second, we found direct evidence that belief alignment increased participants' willingness to act, trust and fairness perception, over and above inconsistent effects of general intuitions about AI itself. That is, outcomes were largely driven by an alignment between AI recommendation and pre‐existing politico‐moral intuitions probed by the scenario contexts, which echoes motivated social cognition needs (Jost, 2017; Jost et al., 2003, 2017; Jost & Amodio, 2012; Jost & Krochik, 2014; Kahan, 2016a, 2016b; Krochik & Jost, 2011; Moore et al., 2021). However, although the effect was evident for all three outcomes in E2, it was only meaningful for willingness to act in E1, possibly because our operationalization of belief alignment did not adequately track the relevance of the deployment contexts (LGBTQ+ rights and environmental protection) to our sample's identities.

While people do have a general political identity that drives affective intuitions (Baldassarri & Page, 2021; Iyengar et al., 2019), they tend to lack ideological coherence (Kalmoe, 2020) and hold moral/political beliefs that diverge from their self‐identified partisan stances on some issues (Smith, 2019). As such, even contentious issues are not monolithic on either end of the political spectrum, and the three‐item scale for overall political position may be inadequate for capturing issue‐specific intuitions probed by the scenarios of AI use. We will address this by using measurements targeted for our topical foci in Experiment 3. Additionally, our measure of fairness did not distinguish between the fairness of the outcome of AI verdicts or involving AI in the process of judgements in these situations. This is akin to the distinction between distributive fairness (Ambrose & Arnaud, 2013) versus procedural fairness (Cropanzano & Ambrose, 2001), which we aim to disentangle in Experiment 3. Lastly, our scenario design attempted to probe a default willingness to initiate an investigation as the direct result of AI verdicts, without explicitly stating it as a consequence of the detected prejudice in the vignettes; we will clarify this in Experiment 3.

## EXPERIMENT 3

We conducted E3 to address the limitations of E1 and E2 outlined above, namely the lack of precision in operationalizing belief (mis)alignment, the entanglement of distributive and procedural fairness concerns, and the unclear implication of AI verdicts of moral transgression for the characters in the vignettes. We directly measured participants' endorsement for specific issues on LGBTQ+ rights and environmental protection in addition to overall political orientation, and modified scenarios and probing questions as appropriate.

Since we operationalize belief alignment via new measures here and as we did not find the predicted belief alignment effects for trust in the AI and perceived fairness of the AI in E1, we only predict: (1) increased willingness to act on AI recommendations as a function of belief alignment and (2) belief alignment will remain over and above general AI attitudes.

### Methods

#### Participants

After removing three participants who failed the attention check, the final data set contains responses collected from 302 native English‐speaking adults (183 males and 116 females; *M*_age_ = 36.83 years, *SD*_age_ = 10.79 years) with no prior involvement in the study. Testing was conducted on Qualtrics via Prolific, for which each subject received £1.20.

#### Design, materials and procedures

Experimental set‐up for E3 was similar to E1, with modifications to the scenario wording, probe questions and measurements of pre‐existing political beliefs. To make explicit the default initiation of investigative actions as the direct consequence of AI detection of potential prejudice, each scenario ended with an extra sentence (‘Based on the AI's recommendation, the court/bank has opened an investigation on this judge/loan manager’). Accordingly, willingness to act on AI recommendations became participant's support to uphold the decision to investigate in line with the AI's verdicts (‘I think that this judge/loan manager should be suspended until the investigation concludes’). This was intended to emphasize a specific action occurring over a simple agreement that some relatively undefined action should occur. The probe question of trust in AI remained unchanged. To better reflect the psychological complexity of fairness judgements, perceived fairness of AI was split into the perception of procedural fairness of using an AI system for such purposes (‘I believe that it's fair to use AI to assess whether a judge/loan manager is biased’) and distributive fairness of the AI verdict itself (‘I believe that the AI's recommendation to investigate is fair’).

Finally, to better capture pre‐existing political positions on our specific scenarios, we added four questions each on LGBTQ+ rights and environmental protection, along with filler items on person‐centred (people of wealth) and cause‐centred (social media) issues as distractions. For each issue, participants responded to a thermometer scale (‘On a scale of 0–100, how warm/cold do you feel about X?’), the extent of concern (‘How concerned are you about X?’; 1 = *not at all concerned* and 5 = *very concerned*) and two more questions on relevant personal experiences (data collected but not analyzed due to a technical error, and pre‐registration was updated prior to analysis; see Appendix B).

#### Statistical analysis plan

Our analyses here are largely identical to E1, with the modification of having four simultaneous dependent variables (willingness to act, trust in AI, perceived procedural fairness, perceived distributive fairness) predicted by fixed effects of context, issue‐specific attitudes and the interaction of the two terms. Participant political position was kept as a covariate to control for its influence on the dependent variables and correlations with issue‐specific attitudes. Other parameters included covariates of GAAIS positive and negative subscale means, nuisance variable of age, as well as random effects of scenario, topic and individual subject. We standardized all variables as appropriate. To obtain continuous measures of issue‐specific attitudes, we averaged the standardized thermometer scale responses and extent of concern of the two topical foci, with higher scores indicating more endorsement for LGBTQ+ rights or environmental protection.

Using the *brms* package (v. 2.19.0; Bürkner, 2017, 2018) in RStudio (v. 4.3.0; R Core Team, 2023), we estimated Bayesian multilevel multivariate multiple regression models containing parameters specified above to predict all four outcomes for each focus separately. Additionally, we explored using overall political position and its interaction with context as main predictors without issue‐specific endorsement to compare with general trends observed in E1. We used the posterior means and *SDs* from E1 (Table 4, top panel) as priors in E3. In cases of absent priors, that is for issue‐specific endorsement and its interaction with context, we used posterior estimates of overall political position and its interaction terms from E1, with signs reversed to indicate greater endorsement for LGBTQ+ rights and environmental protection as the opposite of increasing conservatism.

### Results

#### Descriptive statistics and correlations

Table 5 displays descriptive statistics for E3. Similar to E1 and E2, this sample was largely left‐leaning, with 58.6% scoring an average below four and 13.6% above four on a seven‐point Likert scale across economic, social and foreign policy issues. The overall liberal political orientation was additionally reflected in the reporting of warm feelings and high levels of concern towards LGBTQ+ rights and environmental protection. Closely replicating Schepman and Rodway's (2020, 2022) results once more with both subscales showing good internal consistency (*α*_PosAtt_ = 0.9, *α*_NegAtt_ = 0.83), participants reported positive general attitudes towards practical benefits of AI and held less dystopian concerns over AI. Finally, scenario responses revealed a moderate willingness to uphold investigative actions following AI detection of potential prejudice, and that on average, subjects trusted the AI's recommendation, considered it fair to use AI for such purposes and perceived AI verdict outcomes as fair.

**TABLE 5:** Descriptive summaries of measured variables in Experiments 3.

	Mean (*SD*)	Median	Range
Political positions (1 = *Very Left/Liberal*, 7 = *Very Right/Conservative*)			
Economic issues	3.25 (1.38)	3	6
Social issues	3.05 (1.35)	3	6
Foreign policy issues	3.30 (1.35)	4	6
Mean political position	3.20 (1.28)	3.33	6
Issue‐specific attitudes (higher score indicates positive attitudes)			
LGBTQ+ rights			
Temperate scale (1° ~ 100°)	72.22 (29.97)	82	100
Extent of concern (5‐point Likert scale)	3.40 (1.16)	4	4
Environmental protection			
Temperate scale (1° ~ 100°)	73.55 (22.18)	78	100
Extent of concern (5‐point Likert scale)	4.06 (0.88)	4	4
General attitudes towards AI (5‐point Likert scale; higher score indicates positive attitudes)			
Positive subscale	3.23 (0.63)	3.33	3.75
Negative subscale	3.03 (0.68)	3.12	3.5
Responses to scenarios (1 = *Strongly Disagree*, 5 = *Strongly Agree*)			
Willingness to act	3.15 (1.16)	3.04	4
Trust	3.39 (0.94)	3.49	4
Procedural fairness	3.74 (0.92)	3.99	4
Distributive fairness	3.36 (1.05)	3.53	4

*Note*: For meaningful interpretations, descriptive statistics are presented in original scales.

Table 6 below shows a somewhat different correlational pattern relative to the previous two experiments. First, unsurprisingly, political conservatism showed a moderate negative correlation with endorsement for LGBTQ+ rights and environmental protection, which were moderately positively correlated themselves. Second, positive and reverse‐coded negative GAAIS subscales were positively correlated; while the former was linked to all four outcomes, greater support for LGBTQ+ rights and environmental protection, and less overall conservatism, the latter was linked to most outcomes except willingness to act, and weakly associated with overall political conservatism and endorsement for LGBTQ+ rights. Lastly, all four outcomes showed a positive relationship amongst themselves and with issue‐specific endorsement, although only willingness to act was negatively related to overall political conservatism.

**TABLE 6:** Bayesian Pearson's zero‐order correlations and their 95% HDIs between main variables in Experiment 3.

	Political positions	LGBTQ+ rights	Environmental protection	GAAIS positive	GAAIS negative	Willingness to act	Trust	Procedural fairness
LGBTQ+ rights	−0.46*** [−0.53, −0.41]							
Environmental protection	−0.34*** [−0.41, −0.26]	0.49*** [0.43, 0.55]						
GAAIS positive	−0.12** [−0.19, −0.04]	0.22*** [0.14, 0.29]	0.22*** [0.15, 0.30]					
GAAIS negative	0.08* [0, 0.15]	0.08* [0, 0.16]	−0.04 [−0.12, 0.04]	0.36*** [0.29, 0.43]				
Willingness to act	−0.12*** [−0.20, −0.04]	0.19*** [0.12, 0.27]	0.17*** [0.09, 0.24]	0.11** [0.03, 0.18]	0.04 [−0.04, 0.12]			
Trust	−0.04 [−0.12, 0.04]	0.16*** [0.08, 0.24]	0.27*** [0.20, 0.34]	0.41*** [0.34, 0.47]	0.25*** [0.17, 0.32]	0.46*** [0.39, 0.52]		
Procedural fairness	−0.06 [−0.14, 0.02]	0.18*** [0.11, 0.26]	0.25*** [0.17, 0.32]	0.28*** [0.21, 0.36]	0.16*** [0.07, 0.23]	0.48*** [0.42, 0.54]	0.61*** [0.56, 0.66]	
Distributive fairness	−0.01 [−0.09, 0.07]	0.17*** [0.09, 0.24]	0.25*** [0.18, 0.33]	0.39*** [0.32, 0.45]	0.23*** [0.16, 0.31]	0.35*** [0.28, 0.42]	0.58*** [0.53, 0.63]	0.55*** [0.49, 0.61]

*Note*: Probability of direction (pd) represents the portion of the posterior distribution in the same direction of effect as the median (Makowski et al., 2019). GAAIS negative values are reverse‐coded.

* pd. > 97.5%.

** pd. > 99.5%.

*** pd. > 99.95%.

#### Pre‐registered analysis

**FIGURE 3:**
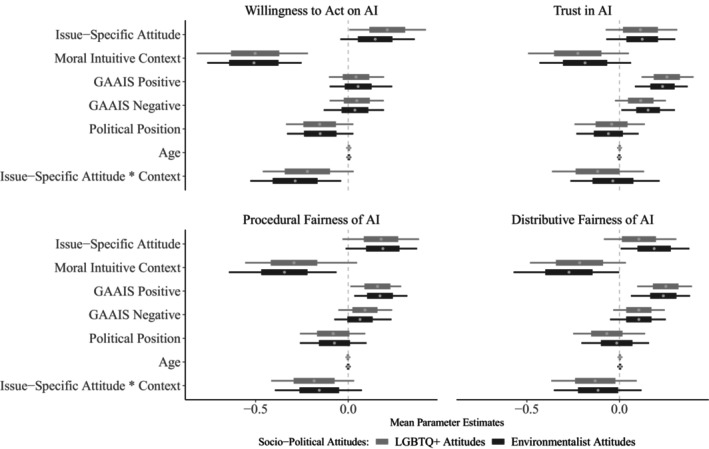
Parameter estimates for Willingness to Act based on AI verdicts, Trust in AI and perception of Procedural Fairness and Distributive Fairness of AI in E3, with boxes indicating 95% HDIs and whiskers indicating 100% HDIs. Higher standardized scores on political position correspond to increasing conservatism. Context is a binary variable with liberal/left‐wing direction as the reference level.

Next, we conducted pre‐registered analyses where issue‐specific attitudes towards LGBTQ+ rights (Table 7a) and environmental protection (Table 7b) were modelled separately while controlling for general political orientation, which produced strikingly similar results (Figure 3). All four outcomes meaningfully increased as a function of increasing individual support for both issues (.11 ≤ all *β*s ≤ .21), but scored lower in the conservative context (−.51 ≤ all *β*s ≤ −.19). Greater positivity towards AI's usefulness predicted greater trust and perception of procedural/distributive fairness across both issues (.17 ≤ all *β*s ≤ .26); lower dystopian AI concerns showed a similar pattern (.09 ≤ all *β*s ≤ .15) except for trust in the environmentalism condition. Ratings for willingness to act stood out, only decreasing with greater participant conservatism (*β*_*a*_ = *β*_*b*_ = −.15).

**TABLE 7:** Summaries of Bayesian regression results in Experiment 3 (pre‐registered models).

	Willingness to act		Trust		Procedural fairness		Distributive fairness	
	Mean [95% HDI]	*SD*	Mean [95% HDI]	*SD*	Mean [95% HDI]	*SD*	Mean [95% HDI]	*SD*
(a) LGBTQ+ rights								
Intercept	0.2 [−0.26, 0.64]	0.23	0.13 [−0.21, 0.54]	0.18	0.3 [−0.04, 0.7]	0.18	0.11 [−0.23, 0.52]	0.18
LGBTQ+ attitudes	**0.21** **[0.11, 0.31]**	0.05	**0.11** **[0.02, 0.21]**	0.05	**0.18** **[0.08, 0.27]**	0.05	**0.11** **[0.01, 0.2]**	0.05
Context	**−0.5** **[−0.63, −0.37]**	0.07	**−0.22** **[−0.35, −0.1]**	0.07	**−0.29** **[−0.42, −0.17]**	0.06	**−0.21** **[−0.34, −0.09]**	0.06
GAAIS positive	0.04 [−0.03, 0.11]	0.04	**0.26** **[0.19, 0.33]**	0.04	**0.16** **[0.09, 0.23]**	0.04	**0.25** **[0.18, 0.32]**	0.04
GAAIS negative	0.05 [−0.02, 0.12]	0.04	**0.12** **[0.05, 0.18]**	0.04	**0.09** **[0.02, 0.16]**	0.04	**0.1** **[0.04, 0.17]**	0.03
Political position	**−0.15** **[−0.24, −0.07]**	0.04	−0.04 [−0.13, 0.04]	0.04	−0.08 [−0.17, 0.01]	0.04	−0.07 [−0.15, 0.02]	0.04
Age	0 [0, 0.01]	0	0 [0, 0.01]	0	0 [−0.01, 0]	0	0 [0, 0.01]	0
LGBTQ+ attitudes × Context	**−0.22** **[−0.34, −0.1]**	0.06	**−0.12** **[−0.24, 0]**	0.06	**−0.18** **[−0.29, −0.07]**	0.06	**−0.13** **[−0.24, −0.02]**	0.06
(b) Environmental concerns								
Intercept	0.06 [−0.3, 0.5]	0.2	0.18 [−0.15, 0.58]	0.18	0.23 [−0.15, 0.68]	0.21	0.06 [−0.29, 0.5]	0.2
Environmentalist attitudes	**0.15** **[0.05, 0.24]**	0.05	**0.12** **[0.04, 0.21]**	0.04	**0.19** **[0.1, 0.28]**	0.05	**0.19** **[0.1, 0.28]**	0.05
Context	**−0.51** **[−0.64, −0.38]**	0.07	**−0.19** **[−0.3, −0.07]**	0.06	**−0.34** **[−0.47, −0.22]**	0.06	**−0.27** **[−0.4, −0.15]**	0.06
GAAIS positive	0.05 [−0.02, 0.13]	0.04	**0.23** **[0.17, 0.3]**	0.03	**0.17** **[0.1, 0.24]**	0.04	**0.24** **[0.17, 0.31]**	0.04
GAAIS negative	0.04 [−0.04, 0.11]	0.04	**0.15** **[0.09, 0.22]**	0.03	0.06 [0, 0.13]	0.03	**0.1** **[0.04, 0.17]**	0.04
Political position	**‐0.15** **[−0.24, −0.06]**	0.04	‐0.06 [−0.14, 0.02]	0.04	−0.07 [−0.16, 0.01]	0.04	−0.01 [−0.1, 0.07]	0.04
Age	0 [0, 0.01]	0	0 [−0.01, 0]	0	0 [−0.01, 0.01]	0	0 [0, 0.01]	0
Env. attitudes × Context	**−0.29** **[−0.41, −0.17]**	0.06	−0.04[−0.14, 0.07]	0.06	**−0.16** **[−0.26, −0.05]**	0.05	**−0.12** **[−0.22, −0.01]**	0.06

*Note*: Model converged successfully with split $\hat{R}$ = 1 for all estimated parameters. Context is a binary variable with liberal/left‐wing direction as the reference level. GAAIS negative values are reverse‐coded. Bold emphasizes 0 ∉ 95% HDI.

Finally, belief (mis)alignment effect was found for both issues, not only for willingness to act as hypothesised (*β*_*a*_ = −.22, 95% HDI = [−0.34, −0.1]; *β*_*b*_ = −.29, 95% HDI = [−0.41, −0.17]), but also for procedural fairness (*β*_*a*_ = −.18, 95% HDI = [−0.29, −0.07]; *β*_*b*_ = −.16, 95% HDI = [−0.26, −0.05]) and distributive fairness (*β*_*a*_ = −.13, 95% HDI = [−0.24, −0.02]; *β*_*b*_ = −.12, 95% HDI = [−0.22, −0.01]), and for trust but only in the LGBTQ+ rights condition (*β*_*a*_ = −.12, 95% HDI = [−0.24, 0]). That is, issue‐specific attitudes and context interacted, such that with conservative AI verdicts, those with greater concern for LGBTQ+ rights and environmental protection were less willing to act on such verdicts, considered involving AI in the decision procedure and the outcome less fair and trusted the AI less.

#### Exploratory analysis

Additional to the pre‐registered analyses, we replicated E1 analysis estimating belief alignment with overall political position without issue‐specific endorsement, returning similar patterns of results: ratings for outcome variables reduce with participant and context conservatism and increase with greater positivity towards AI. Unlike E1, however, belief alignment was meaningful for all outcomes (.1 ≤ all *β*s ≤ .21; see Appendix E.1). We also explored three‐way interactions between context, issue‐specific/overall political positions and positive/negative general AI attitudes; as in E1 & E2, the more complex model specification did not improve predictive power, again strongly favouring simpler models (see Appendix E.4).

### Discussion

E3 generated three main results. First, as in E1 and E2, participants' willingness to act on AI verdicts and judgements of trust and fairness was higher in the liberal context and stronger as a function of increasing support for LGBTQ+ rights and environmental protection; unsurprising, given this sample was, again, left‐leaning and reported strong endorsement for both issues.

Second, positivity towards AI predicted greater trust and perceptions of both procedural and distributive fairness. This may stem from the presentation of AI verdicts as purely statistical patterns, giving the impression that prejudice, a morally charged phenomenon reduced to statistical anomalies, is measurable and quantifiable. In line with previous findings of task‐specific algorithmic aversion towards delegating to AI perceived subjective tasks (Castelo et al., 2019; Lee, 2018), our results suggest that the perception of AI use in moral situations commonly considered to require unique human abilities, such as freewill and autonomy (Jauernig et al., 2022), may be improved by the perceived objectivity of the task at hand.

Third and most importantly, we reproduced belief alignment for both overall political position and specific endorsement for LGBTQ+ rights and environmental protection (only except trust in the environmentalism condition). This suggests judgements towards AI verdicts of moral transgression were in part driven by the alignment of the verdict with participants' pre‐existing politico‐moral beliefs in those contexts, which are themselves correlated with overall political leaning. The consistent pattern of belief alignment here across overall political orientation and specific endorsement eased our previous concern that general political position might be inadequate at capturing issue‐specific intuitions probed by our chosen contexts. Furthermore, general AI attitudes were as strong as, if not stronger than, belief alignment itself for trust and fairness judgements, but not for willingness to act. This suggests a possible tension that despite judging the AI and its output as trustworthy and/or fair, participants were not always willing to act on its verdicts. Aydin and Malle (2024) report a similar disassociation between processes of deliberating the content of the advice and assessing the characteristics of advisors (e.g. trustworthiness). They found that while the persuasion effect of both AI and human legal advisors was equally strong, human advisors received greater approval and trust, compared to AI advisors. These findings highlight the nuances involved in AI‐mediated moral judgements that require further research.

## GENERAL DISCUSSIONS

### Summary of main findings

Using morally charged vignettes, we investigated whether participants' judgements towards AI decisions are driven by their belief alignment with the underlying context of AI deployment over and above general AI attitudes. Across three experiments, we found evidence that (1) while positivity about AI influenced judgements towards its deployment (especially for judgements of trust and fairness), they were also uniquely driven by the compatibility between AI verdicts and participants' existing moral/political values beyond general attitudes towards AI itself; (2) these effects were often in tension with one another and of similar magnitude; and that (3) liberal‐leaning orientations and contexts predicted more willingness to act on AI verdicts, more trust and greater perception of (procedural/distributive) fairness. These results offer several theoretical implications for judgement and decision‐making and practical implications for the future adoption of automated decision‐making in morally significant contexts.

### Implications, limitations and further research

First, the finding of belief alignment is consistent with research on motivated social cognition where individuals have the tendency to selectively accept/process belief‐consistent information or intuitively reject belief‐inconsistent information (e.g. Lewandowsky & Oberauer, 2016; Moore et al., 2021; Tucker et al., 2018). Since much research has been conducted on politicized social/scientific beliefs (e.g. Drummond & Fischhoff, 2017; Glinitzer et al., 2021), we provide an example of motivated reasoning in human–computer interaction. It is noteworthy that belief alignment effects are not necessarily indicative of bias and can at times reflect rational information processing principles (Cook & Lewandowsky, 2016). Furthermore, both theoretical simulations (Hahn et al., 2020) and behavioural experiments (Fränken et al., 2020, 2024) suggest that inference on social information does not account for the structure of information dependency – people do not appear to distinguish hearsay from evidence, and trust may play a significant role in moderating the force of such socially driven learning. Thus, our results that positive AI attitudes predict increased trust in, perceived fairness of, and occasionally willingness to act on AI verdicts of human transgression can act as a counterweight to belief misalignment.

The increase in trust and fairness judgements, driven by general optimism towards AI, may pose risks of misuse by corporations or governments seeking to further their own interests (Liu et al., 2022). This is further complicated by the fact that the consultation, delegation or deference of moral judgements to AI can introduce complications in assigning responsibility and blame/praise for multi‐agent decision‐making (Matthias, 2004; Vallor & Vierkant, 2024). Further research should investigate how individuals assign responsibility, blame and credit when they follow or disregard AI suggestions as a result of belief (mis) alignment. Initial research on the attribution of responsibility involving AI advisors has already shown that compatibility between AI suggestions/advice and one's (meta) desires/intentions could licence a particular decision/behaviour, which might in turn lead to the transfer of responsibility to the enabling agent (Dong & Bocian, 2024). Amidst the increasingly ubiquitous applications of AI, this is particularly crucial for technologies with the potential to profoundly influence many lives, such as medical diagnosis, transplant allocation, loan outcomes and admissions or employment decisions.

Moreover, trust and fairness perception, while positively correlated with attitudes towards the practical benefits of AI, are not reducible to willingness to uphold AI decisions. Fairness perceptions of algorithmic decision outcomes and processes are conceptually linked and have been studied extensively: algorithmic factors such as accuracy, transparency and input features all influence perceived fairness, along with human factors like socio‐demographics, self‐interest and familiarity (Starke et al., 2022). Trust itself can be separated into functionality‐based and human‐like trust in AI (Choung et al., 2023), with the former potentially linking to competence and reliability (ability‐based performance trust) and the latter to sincerity, integrity and benevolence (human‐like moral trust) (Malle & Ullman, 2021; see also Ullman & Malle, 2018, 2019). This aligns with previous findings highlighting an objective–subjective distinction in task characteristics, where tasks with more quantifiable aspects tend to elicit higher levels of trust (Castelo et al., 2019; Lee, 2018). Future studies could clarify the relationships between these different facets of trust across different types of AI applications. However, both fields of study reveal inconclusive results concerning the factors influencing trust(worthiness) and the perception of AI, as well as human–AI comparative effects. It is thus crucial to recognize context dependency in trust and fairness in algorithmic decisions such that a general conclusion across board is likely infeasible.

We further found that despite the prevalent aversion for involving AI in moral decision‐making, participants exhibited positive attitudes towards this particular form of algorithmic detection of prejudice. A possible explanation for this could be the reduction of moral transgression (e.g. prejudice) to a simple statistical pattern of favouring/discriminating against particular groups of individuals (e.g. loan application success rate for LGBTQ+ couples). This presentation of prejudice identification as an objective and/or quantifiable task may have somewhat mitigated algorithmic aversion in the moral domain. Considering the inherent morally relevant features in many contexts of AI deployment, however, while this may enhance the acceptability of algorithms, overly reductionistic computerisation of intricate moral matters could lead to long‐term harm if fundamental structural issues underpinning, for example, prejudice, remain neglected. This concern is heightened by the rise of popular generative AI technologies, particularly following the release of ChatGPT in 2022: given the ease with which these systems can generate and disseminate false or misleading information, presenting AI‐generated content as objective ‘ground truth’ is potentially dangerous and raises significant ethical challenges.

Methodologically, our characterization of a narrow statistical pattern‐detection AI is both a limitation and a strength. It is constrained in scope and somewhat outdated, particularly in comparison to more recent advancements, such as large language models (LLMs) that involve more sophisticated deep learning techniques, trained on vast amounts of data and refined through reinforcement learning from human feedback. A recent large‐scale study (*N* = 4836) has already shown that LLM‐generated messages on political policies are just as persuasive as human‐written ones, even for highly polarized topics such as gun control (Bai et al., 2023). As such, further research is needed to explore the impact of belief alignment on more human‐like, assertive language coming from AI, given the well‐documented risks of such models generating misinformation and nonsensical content (Hicks et al., 2024).

Yet, our description of a rigid, non‐agentic AI allows us to isolate the role of belief alignment motivated by underlying contexts of AI deployment. Finding this effect as strong as general AI attitudes towards a simple, static AI speaks to how much more pronounced this effect might be with more complex systems and more information available regarding how they are built, trained, by whom and for what purposes. Given the known and widely reported algorithmic bias (Gebru, 2020; Mehrabi et al., 2021; Weidinger et al., 2022) and growing public concerns towards big tech companies (Ibrahim et al., 2024), such assumptions may amplify the motivated cognition aspects of judgements towards AI output, potentially enhancing the magnitude of belief alignment, especially through feeding back into the training data (Kidd & Birhane, 2023).

Finally, those more familiar or experienced with algorithmic/AI justice literature or technical aspects of AI may interpret our stimuli differently from the general public; we also cannot be certain if participants made other assumptions unaccounted for by our materials. As a result, GAAIS may be insufficient for capturing more nuanced attitudes towards AI, as it may not effectively differentiate between more subtle perspectives or various types of AI applications. While it is adequate for our purposes of gauging general sentiments towards AI amongst the broader public, future research could explore this issue further, potentially adopting a mixed‐methods approach that incorporates qualitative data.

## CONCLUSIONS

In this three‐part pre‐registered study, we examined how the alignment of prior socio‐moral beliefs with contextual information impacted judgement on AI moral recommendations. Results revealed that both belief alignment and general positivity towards AI influenced willingness to act on AI verdicts, trust in AI and perceived procedural/distributive fairness of AI. As such, previous research on motivated reasoning extends to artificial agents, highlighting the complexity of AI‐mediated moral judgements given the emergence of AI as a category of pseudo‐moral agents and potential risks in the large‐scale deployment of autonomous decision systems, even with human oversight.

## AUTHOR CONTRIBUTIONS

**Yuxin Liu:** Conceptualization; methodology; software; data curation; investigation; validation; formal analysis; funding acquisition; visualization; project administration; resources; writing – original draft; writing – review and editing. **Adam Moore:** Conceptualization; formal analysis; methodology; investigation; software; supervision; writing – review and editing.

## CONFLICT OF INTEREST STATEMENT

The authors declare no conflicts of interest.

## Supporting information


Data S1

## Data Availability

The data that support the findings of this study are openly available on Open Science Framework at https://osf.io/7qjt3/.
