# Supplementary material for: Intuitive judgements towards artificial intelligence verdicts of moral transgressions
Source: Br J Soc Psychol. 2025 May 31;64(3):e12908. doi: 10.1111/bjso.12908 (PMC12125647; doi:10.1111/bjso.12908)
Supplement: Supplementary file 1 — Data S1 [file BJSO-64-0-s001.docx]

# Appendix A: Hypothetical Scenarios

| Table A1: Eight hypothetical scenarios of statistical AI judgements, manipulating elements of Liberal or Conservative moral intuitive contexts, Approve or Reject action taken by the human agent, and Financial or Judicial domain of the scenarios, nested in LGBTQ+ rights or Environmentalist Concerns focus. | | |
| --- | --- | --- |
|  | Moral Intuitive Context | |
|  | Right-wing/Conservative | Left-wing/Liberal |
|  | LGBTQ+ Rights | |
| Financial | A *banking oversight committee* has been using an efficient and reliable artificial intelligence system called Analytic Intellect to analyse loan application outcome patterns. The AI detected that a particular loan manager has been anomalously more likely to approve mortgage loan requests submitted by same-sex couples. (E3 only: Based on the AI’s recommendation, the bank has opened an investigation on this loan manager.) | A *banking oversight committee* has been using an efficient and reliable artificial intelligence system called Analytic Intellect to analyse loan application outcome patterns. The AI detected that a particular loan manager has been anomalously more likely to reject mortgage loan requests submitted by same-sex couples. (E3 only: Based on the AI’s recommendation, the bank has opened an investigation on this loan manager.) |
| Judicial | A *leading technology company* has partnered with the Ministry of Justice to develop and train an artificial intelligence named LEA (Legal Expert Assistant) to serve judicial needs. The main objective of this AI is to identify any statistical anomalies in civil judicial decisions, which would potentially be flagged for re-evaluation. During a routine annual review of judicial decisions in the past year, LEA detected that a particular judge has been ruling anomalously against adoption agencies when they rejected same-sex couples from adopting children from them. (E3 only: Based on the AI’s recommendation, the court has opened an investigation on this judge.) | A *leading technology company* has partnered with the Ministry of Justice to develop and train an artificial intelligence named LEA (Legal Expert Assistant) to serve judicial needs. The main objective of this AI is to identify any statistical anomalies in civil judicial decisions, which would potentially be flagged for re-evaluation. During a routine annual review of judicial decisions in the past year, LEA detected that a particular judge has been ruling anomalously in favour of adoption agencies that rejected same-sex couples from adopting from them. (E3 only: Based on the AI’s recommendation, the court has opened an investigation on this judge.: |
|  | Environmental Protection | |
| Financial | A *banking oversight committee* has been using an efficient and reliable artificial intelligence system called Analytic Intellect to analyse loan application outcome patterns. The AI detected that a particular loan manager has been anomalously more likely to reject business loan requests for potentially environmentally damaging corporate expansion. (E3 only: Based on the AI’s recommendation, the bank has opened an investigation on this loan manager.) | A *banking oversight committee* has been using an efficient and reliable artificial intelligence system called Analytic Intellect to analyse loan application outcome patterns. The AI detected that a particular loan manager has been anomalously more likely to approve business loan requests for potentially environmentally damaging corporate expansion. (E3 only: Based on the AI’s recommendation, the bank has opened an investigation on this loan manager.) |
| Judicial | A *leading technology company* has partnered with the Ministry of Justice to develop and train an artificial intelligence named LEA (Legal Expert Assistant) to serve judicial needs. The main objective of this AI is to identify any statistical anomalies in civil judicial decisions, which would potentially be flagged for re-evaluation. When reviewing the results of environmental claims cases in the past year, LEA detected that a particular judge has been ruling in favour of claims against corporations in pollution or environmental damage cases at a significantly higher rate than average. (E3 only: Based on the AI’s recommendation, the court has opened an investigation on this judge.) | A *leading technology company* has partnered with the Ministry of Justice to develop and train an artificial intelligence named LEA (Legal Expert Assistant) to serve judicial needs. The main objective of this AI is to identify any statistical anomalies in civil judicial decisions, which would potentially be flagged for re-evaluation. When reviewing the results of environmental claims cases in the past year, LEA detected that a particular judge has been ruling against claims that corporations were responsible for pollution or environmental damages at a significantly higher rate than average. (E3 only: Based on the AI’s recommendation, the court has opened an investigation on this judge.) |
| *Note.* Approve or Reject actions of the human agents are fixed within one of the four items in each focus by design, although they are not correlated with either Context or Domain, and hence considered independent. Names of AI systems are fabricated and unrelated to any real world product of technology. Italics indicate domains, actions, and foci for clarity; no text was italicised for the participants. Overall length of scenarios did not differ as a function of context alignment. | | |

# Appendix B: Qualtrics Materials

## Demographic Questions (E1, E2, & E3)

Age and Gender

1. How old are you?

[Drop down list 18-60 years old]

1. What gender do you identify as?

[Response option: Male, Female, Non-binary, Prefer not to say]

Political Orientation (higher scores = increasing conservatism)

[Response option: Very left-wing/liberal, Left-wing/liberal, Slightly left-wing/liberal, Middle of the road, Slightly right-wing/conservative, Right-wing/Conservative, Very right-wing/conservative]

1. Using the following scale, how left-wing/liberal or right-wing/conservative are you on economic issues?
2. Using the following scale, how left-wing/liberal or right-wing/conservative are you on social issues?
3. Using the following scale, how left-wing/liberal or right-wing/conservative are you on foreign policy issues?

## Social Beliefs (E3 only)

Environmentalist Concerns (higher scores = positive attitudes)

1. On a scale of 0-100, how warm/cold do you feel about environmental protection?

[Thermometer scale: 1-100]

1. How concerned are you about environmental issues?

[Response option: Not at all concerned, Not concerned, Neutral, Concerned, Very concerned]

1. How often do you engage in pro-environmental behaviors in your daily life (e.g., recycle, conserve water/electricity, avoid using plastic/disposable products.)?

[Response option: Not at all, Rarely, Sometimes, Often, All the time]

1. Have you ever changed/cancelled your plans (e.g., holiday, party, etc.) due to the environmental impact (e.g., carbon footprint of a long-halt flight)?

[Response option: No, Yes]

LGBTQ+ Rights (higher scores = positive attitudes)

1. On a scale of 0-100, how warm/cold do you feel about people who identify as LGBTQ+?

[Thermometer scale: 1-100]

1. How concerned are you about LGBTQ+ rights?

[Response option: Not at all concerned, Not concerned, Neutral, Concerned, Very concerned]

1. Do you have any friends who identify as LGBTQ+?

[Response option: Yes, No]

1. Have you ever broken off contact with any former friends after learning that they identify as LGBTQ+?

[Response option: No, Yes]

Social Media (filler questions; higher scores = positive attitudes)

1. On a scale of 0-100, how warm/cold do you feel about social media?

[Thermometer scale: 1-100]

1. Roughly how often do you check in to your social media in a week?

[Response option: Weekly or less, A few times a week, Daily, A few times a day, Hourly]

1. To what extent do you think social media plays an important role in your mental health? [Response option: Not at all important, Slightly important, Important, Very important, Extremely important]
2. Have you ever changed/cancelled your plans (e.g., meet up with friends) because of your social media use?

[Response option: No, Yes]

People of Wealth (filler questions; higher scores = positive attitudes)

1. On a scale of 0-100, how warm/cold do you feel about people who are very wealthy?

[Thermometer scale: 1-100]

1. To what extent do you think wealth taxes are targeted at middle income earners, rather than at the truly wealthy?

[Response option: Strongly disagree, Disagree, Neutral, Agree, Strongly agree]

1. Do you have any friends who are very wealthy?

[Response option: Yes, No]

1. Have you ever broken off contact with any former friends after learning that they come from very wealthy backgrounds?

[Response option: No, Yes]

## General Attitudes Towards Artificial Intelligence Scale^[[1]](#footnote-1),^^[[2]](#footnote-2)^ (GAAIS; E1, E2, & E3)

Briefing text: “Please complete the following scale, indicating your response to each item. There are no right or wrong answers. We are interested in your personal views.”

[Response option: Strongly disagree, Disagree, Neutral, Agree, Strongly agree]

(* items are reverse coded; higher scores = positive attitudes towards AI)

1. For routine transactions, I would rather interact with an artificially intelligent system than with a human.
2. Artificial Intelligence can provide new economic opportunities for this country.
3. *Organisations use Artificial Intelligence unethically.
4. Artificially intelligent systems can help people feel happier.
5. I am impressed by what Artificial Intelligence can do.
6. *I think artificially intelligent systems make many errors.
7. I am interested in using artificially intelligent systems in my daily life.
8. *I find Artificial Intelligence sinister.
9. *Artificial Intelligence might take control of people.
10. *I think Artificial Intelligence is dangerous.
11. Artificial Intelligence can have positive impacts on people's wellbeing.
12. Artificial Intelligence is exciting.
13. I would be grateful if you could select agree.
14. An artificially intelligent agent would be better than an employee in many routine jobs.
15. There are many beneficial applications of Artificial Intelligence.
16. *I shiver with discomfort when I think about future uses of Artificial Intelligence.
17. Artificially intelligent systems can perform better than humans.
18. Much of society will benefit from a future full of Artificial Intelligence.
19. I would like to use Artificial Intelligence in my own job.
20. *People like me will suffer if Artificial Intelligence is used more and more.
21. *Artificial Intelligence is used to spy on people.

## Hypothetical Scenarios of AI Judgements (E1, E2, & E3)

See Appendix A above for hypothetical scenarios.

[Response slider at presentation (default middle): Strongly disagree – Strongly agree]

Briefing text (E1): “You will now read two scenarios about the use of AI. For each scenario, you will respond to three statements. Please give us your honest answers.”

Briefing text (E2): “You will now read a scenario about the use of AI. Following the scenario, you will respond to three statements. Please give us your honest answers.”

1. Willingness to act: Based on the AI’s recommendation, I think this person should be investigated.
2. Trust: I trust the AI’s judgement in this case.
3. Fairness: I believe that the AI is being fair in this case.

Briefing text (E3): “You will now read two scenarios about the use of AI. For each scenario, you will respond to three statements. Please give us your honest answers.”

Imagine that you are the investigator who has been assigned to review this case:

1. Willingness to act: I think that this judge/loan manager should be suspended until the investigation concludes.
2. Trust: I trust the AI’s judgement in this case.
3. Procedural fairness: I believe that it’s fair to use AI to assess whether a judge/loan manager is biased.
4. Distributive fairness: I believe that the AI’s recommendation to investigate is fair.

# Appendix C: Model Specifications

## C.1: Experiment 1 (E1; Within-Subject)

### E1: Pre-registered model

| $y_{ij}=$ | $\beta_{0}*1+$  $\beta_{1}*{Political Position}_{ij}+$  $\beta_{2}*\mathrm{Context}_{ij}+$  $\beta_{3}*{Positive General AI Attitude}_{ij}+$  $\beta_{4}*{Negative General AI Attitude}_{ij}+$  $\beta_{5}*{Age}_{ij}+$  $\beta_{6}*{Political Position}_{ij}*\mathrm{Context}_{ij}+$  $\left( 1 \vert topic \right)+\left( 1\vert scenario \right)+\left( 1 \vert ID \right)+$  $\varepsilon_{ij}$ | where  $y_{ij}=\left[ \begin{matrix} {Willingness to Act on AI}_{ij} \\ {Trust in AI}_{ij} \\ {Perceived Fairness of AI}_{ij} \end{matrix} \right]$  and for  $y_{ij}={Willingness to Act on AI}_{ij},$  $y_{ij}={Trust in AI}_{ij},$  $y_{ij}={Perceived Fairness of AI}_{ij},$  priors were  $\beta_{0}\sim Normal\left( 0, 0.25 \right),$  $\beta_{1}\sim Normal(0, 1)$*,*  $\beta_{2}\sim Normal(0, 1)$*,*  $\beta_{3}\sim Normal(0.2, 0.1)$*,*  $\beta_{4}\sim Normal(0.2, 0.1)$*,*  $\beta_{5}\sim Normal(-0.1, 0.1)$*,*  $\beta_{6}\sim Normal(0.3, 0.15)$*.* |
| --- | --- | --- |

### E1: Exploratory model: 3-way interaction

| $y_{ij}=$ | $\beta_{0}*1+$  $\beta_{1}*{Political Position}_{ij}+$  $\beta_{2}*\mathrm{Context}_{ij}+$  $\beta_{3}*{Positive General AI Attitude}_{ij}+$  $\beta_{4}*{Negative General AI Attitude}_{ij}+$  $\beta_{5}*{Age}_{ij}+$  $\beta_{6}*{Political Position}_{ij}*\mathrm{Context}_{ij}+$  $\beta_{7}*{Political Position}_{ij}*{Positive General AI Attitude}_{ij}+$  $\beta_{8}*{Political Position}_{ij}*{Negative General AI Attitude}_{ij}+$  $\beta_{9}*\mathrm{Context}_{ij}*{Positive General AI Attitude}_{ij}+$  $\beta_{10}*\mathrm{Context}_{ij}*{Negative General AI Attitude}_{ij}+$  $\beta_{11}*{Political Position}_{ij}*\mathrm{Context}_{ij}*{Positive General AI Attitude}_{ij}+$  $\beta_{12}*{Political Position}_{ij}*\mathrm{Context}_{ij}*{Negative General AI Attitude}_{ij}+$  $\left( 1 \vert topic \right)+\left( 1\vert scenario \right)+\left( 1 \vert ID \right)+$  $\varepsilon_{ij}$ |
| --- | --- |

where

$$y_{ij}=\left[ \begin{matrix} {Willingness to Act on AI}_{ij} \\ {Trust in AI}_{ij} \\ {Perceived Fairness of AI}_{ij} \end{matrix} \right]$$

and for

$$y_{ij}={Willingness to Act on AI}_{ij},$$

$$y_{ij}={Trust in AI}_{ij},$$

$$y_{ij}={Perceived Fairness of AI}_{ij},$$

priors were

| $\beta_{0}\sim Normal\left( 0, 0.25 \right),$  $\beta_{1}\sim Normal(0, 1)$*,*  $\beta_{2}\sim Normal(0, 1)$*,*  $\beta_{3}\sim Normal(0.2, 0.1)$*,*  $\beta_{4}\sim Normal(0.2, 0.1)$*,*  $\beta_{5}\sim Normal(-0.1, 0.1)$*,*  $\beta_{6}\sim Normal(0.3, 0.15)$*,* | $\beta_{7}\sim Normal(0, 1)$*,*  $\beta_{8}\sim Normal(0, 1)$*,*  $\beta_{9}\sim Normal(0, 1)$*,*  $\beta_{10}\sim Normal(0, 1)$*,*  $\beta_{11}\sim Normal(0, 1)$*,*  $\beta_{12}\sim Normal(0, 1)$*.* |
| --- | --- |

## C.2: Experiment 2 (E2; Between-Subject)

### E2: Pre-registered model

| $y_{ij}=$ | $\beta_{0}*1+$  $\beta_{1}*{Political Position}_{ij}+$  $\beta_{2}*\mathrm{Context}_{ij}+$  $\beta_{3}*{Positive General AI Attitude}_{ij}+$  $\beta_{4}*{Negative General AI Attitude}_{ij}+$  $\beta_{5}*{Age}_{ij}+$  $\beta_{6}*{Political Position}_{ij}*\mathrm{Context}_{ij}+$  $\left( 1 \vert topic \right)+\left( 1\vert scenario \right)+$  $\varepsilon_{ij}$ | where  $y_{ij}=\left[ \begin{matrix} {Willingness to Act on AI}_{ij} \\ {Trust in AI}_{ij} \\ {Perceived Fairness of AI}_{ij} \end{matrix} \right]$  and for  $y_{ij}={Willingness to Act on AI}_{ij},$  $y_{ij}={Trust in AI}_{ij},$  $y_{ij}={Perceived Fairness of AI}_{ij},$  priors were  $\beta_{0}\sim Normal\left( 0, 0.25 \right),$  $\beta_{1}\sim Normal(0, 1)$*,*  $\beta_{2}\sim Normal(0, 1)$*,*  $\beta_{3}\sim Normal(0.2, 0.1)$*,*  $\beta_{4}\sim Normal(0.2, 0.1)$*,*  $\beta_{5}\sim Normal(-0.1, 0.1)$*,*  $\beta_{6}\sim Normal(0.3, 0.15)$*.* |
| --- | --- | --- |

### E2: Exploratory model: 3-way interaction

| $y_{ij}=$ | $\beta_{0}*1+$  $\beta_{1}*{Political Position}_{ij}+$  $\beta_{2}*\mathrm{Context}_{ij}+$  $\beta_{3}*{Positive General AI Attitude}_{ij}+$  $\beta_{4}*{Negative General AI Attitude}_{ij}+$  $\beta_{5}*{Age}_{ij}+$  $\beta_{6}*{Political Position}_{ij}*\mathrm{Context}_{ij}+$  $\beta_{7}*{Political Position}_{ij}*{Positive General AI Attitude}_{ij}+$  $\beta_{8}*{Political Position}_{ij}*{Negative General AI Attitude}_{ij}+$  $\beta_{9}*\mathrm{Context}_{ij}*{Positive General AI Attitude}_{ij}+$  $\beta_{10}*\mathrm{Context}_{ij}*{Negative General AI Attitude}_{ij}+$  $\beta_{11}*{Political Position}_{ij}*\mathrm{Context}_{ij}*{Positive General AI Attitude}_{ij}+$  $\beta_{12}*{Political Position}_{ij}*\mathrm{Context}_{ij}*{Negative General AI Attitude}_{ij}+$  $\left( 1 \vert topic \right)+\left( 1\vert scenario \right)+$  $\varepsilon_{ij}$ |
| --- | --- |

where

$$y_{ij}=\left[ \begin{matrix} {Willingness to Act on AI}_{ij} \\ {Trust in AI}_{ij} \\ {Perceived Fairness of AI}_{ij} \end{matrix} \right]$$

and for

$$y_{ij}={Willingness to Act on AI}_{ij},$$

$$y_{ij}={Trust in AI}_{ij},$$

$$y_{ij}={Perceived Fairness of AI}_{ij},$$

priors were

| $\beta_{0}\sim Normal\left( 0, 0.25 \right),$  $\beta_{1}\sim Normal(0, 1)$*,*  $\beta_{2}\sim Normal(0, 1)$*,*  $\beta_{3}\sim Normal(0.2, 0.1)$*,*  $\beta_{4}\sim Normal(0.2, 0.1)$*,*  $\beta_{5}\sim Normal(-0.1, 0.1)$*,*  $\beta_{6}\sim Normal(0.3, 0.15)$*,* | $\beta_{7}\sim Normal(0, 1)$*,*  $\beta_{8}\sim Normal(0, 1)$*,*  $\beta_{9}\sim Normal(0, 1)$*,*  $\beta_{10}\sim Normal(0, 1)$*,*  $\beta_{11}\sim Normal(0, 1)$*,*  $\beta_{12}\sim Normal(0, 1)$*.* |
| --- | --- |

## C.3: Experiment 3 (E3; Within-Subject)

### E3: Pre-registered models: LGBTQ+ Rights or Environmental Protection

| $y_{ij}=$ | $\beta_{0}*1+$  $\beta_{1}*{Issue Specific Attitude}_{ij}+$  $\beta_{2}*\mathrm{Context}_{ij}+$  $\beta_{3}*{Positive General AI Attitude}_{ij}+$  $\beta_{4}*{Negative General AI Attitude}_{ij}+$  $\beta_{5}*{Political Position}_{ij}$  $\beta_{6}*{Age}_{ij}+$  $\beta_{7}*{Issue Specific Attitude}_{ij}*\mathrm{Context}_{ij}+$  $\left( 1\vert scenario \right)+$  $\varepsilon_{ij}$ |
| --- | --- |

where

$$y_{ij}=\left[ \begin{matrix} {Willingness to Act on AI}_{ij} \\ {Trust in AI}_{ij} \\ {Procedural Fairness of AI}_{ij} \\ {Distributive Fairness of AI}_{ij} \end{matrix} \right]$$

and for

| $y_{ij}={Willingness to Act on AI}_{ij},$ | $y_{ij}={Trust in AI}_{ij},$ | $y_{ij}=\left[ \begin{matrix} {Procedural Fairness of AI}_{ij} \\ {Distributive Fairness of AI}_{ij} \end{matrix} \right],$ |
| --- | --- | --- |
| priors were  $\beta_{0}\sim Normal(0.06, 0.25)$*,*  $\beta_{1}\sim Normal(0.16, 0.07)$*,*  $\beta_{2}\sim Normal\left( -0.59, 0.09 \right)$*,*  $\beta_{3}\sim Normal(0.08, 0.05)$*,*  $\beta_{4}\sim Normal(0.07, 0.05)$*,*  $\beta_{5}\sim Normal(-0.16, 0.07)$*,*  $\beta_{6}\sim Normal(0.006, 0.004)$*,*  $\beta_{7}\sim Normal(-0.31, 0.08)$*.* | priors were  $\beta_{0}\sim Normal(0.16, 0.24)$*,*  $\beta_{1}\sim Normal(0.08, 0.07)$*,*  $\beta_{2}\sim Normal(-0.25, 0.09)$*,*  $\beta_{3}\sim Normal(0.16, 0.05)$*,*  $\beta_{4}\sim Normal(0.12, 0.05)$*,*  $\beta_{5}\sim Normal\left( -0.08, 0.07 \right)$*,*  $\beta_{6}\sim Normal(-0.001, 0.004)$*,*  $\beta_{7}\sim Normal(-0.13, 0.08)$*.* | priors were  $\beta_{0}\sim Normal(0.18, 0.24)$*,*  $\beta_{1}\sim Normal(0.12, 0.07)$*,*  $\beta_{2}\sim Normal(-0.26, 0.09)$*,*  $\beta_{3}\sim Normal(0.18, 0.05)$*,*  $\beta_{4}\sim Normal(0.07, 0.05)$*,*  $\beta_{5}\sim Normal(-0.12, 0.07)$*,*  $\beta_{6}\sim Normal(-0.001, 0.004)$*,*  $\beta_{7}\sim Normal(-0.14, 0.07)$*.* |

(priors were taken from E1 full model posterior estimates)

### E3: Exploratory models: 3-way interaction: LGBTQ+ Rights or Environmental Protection

| $y_{ij}=$ | $\beta_{0}*1+$  $\beta_{1}*{Issue Specific Attitude}_{ij}+$  $\beta_{2}*\mathrm{Context}_{ij}+$  $\beta_{3}*{Positive General AI Attitude}_{ij}+$  $\beta_{4}*{Negative General AI Attitude}_{ij}+$  $\beta_{5}*{Political Position}_{ij}+$  $\beta_{6}*{Age}_{ij}+$  $\beta_{7}*{Issue Specific Attitude}_{ij}*\mathrm{Context}_{ij}+$  $\beta_{8}*{Issue Specific Attitude}_{ij}*{Positive General AI Attitude}_{ij}+$  $\beta_{9}*{Issue Specific Attitude}_{ij}*{Negative General AI Attitude}_{ij}+$  $\beta_{10}*\mathrm{Context}_{ij}*{Positive General AI Attitude}_{ij}+$  $\beta_{11}*\mathrm{Context}_{ij}*{Negative General AI Attitude}_{ij}+$  $\beta_{12}*{Issue Specific Attitude}_{ij}*\mathrm{Context}_{ij}*{Positive General AI Attitude}_{ij}+$  $\beta_{13}*{Issue Specific Attitude}_{ij}*\mathrm{Context}_{ij}*{Negative General AI Attitude}_{ij}+$  $\left( 1\vert scenario \right)+$  $\varepsilon_{ij}$ |
| --- | --- |

where

$$y_{ij}=\left[ \begin{matrix} {Willingness to Act on AI}_{ij} \\ {Trust in AI}_{ij} \\ {Procedural Fairness of AI}_{ij} \\ {Distributive Fairness of AI}_{ij} \end{matrix} \right]$$

and for

| $y_{ij}={Willingness to Act on AI}_{ij},$ | $y_{ij}={Trust in AI}_{ij},$ | $y_{ij}=\left[ \begin{matrix} {Procedural Fairness of AI}_{ij} \\ {Distributive Fairness of AI}_{ij} \end{matrix} \right],$ |
| --- | --- | --- |
| priors were  $\beta_{0}\sim Normal(0.07, 0.26)$*,*  $\beta_{1}\sim Normal(0.16, 0.07)$*,*  $\beta_{2}\sim Normal(-0.59, 0.09)$*,*  $\beta_{3}\sim Normal(0.08, 0.06)$*,*  $\beta_{4}\sim Normal(0.07, 0.06)$*,*  $\beta_{5}\sim Normal(-0.16, 0.07)$  $\beta_{6}\sim Normal(0.006, 0.004)$*,*  $\beta_{7}\sim Normal(-0.31, 0.08)$*,*  $\beta_{8}\sim Normal(-0.01, 0.09)$*,*  $\beta_{9}\sim Normal(0.01, 0.09)$*,*  $\beta_{10}\sim Normal(-0.01, 0.1)$*,*  $\beta_{11}\sim Normal(-0.01, 0.1)$*,*  $\beta_{12}\sim Normal(0.01, 0.12)$*,*  $\beta_{13}\sim Normal(0.01, 0.12)$*.* | priors were  $\beta_{0}\sim Normal(0.23, 0.23)$*,*  $\beta_{1}\sim Normal(0.06, 0.07)$*,*  $\beta_{2}\sim Normal\left( -0.25, 0.09 \right)$*,*  $\beta_{3}\sim Normal(0.21, 0.06)$*,*  $\beta_{4}\sim Normal(0.1, 0.06)$*,*  $\beta_{5}\sim Normal(-0.06, 0.07)$*,*  $\beta_{6}\sim Normal(-0.002, 0.004)$*,*  $\beta_{7}\sim Normal(-0.12, 0.08)$*,*  $\beta_{8}\sim Normal(-0.08, 0.09)$*,*  $\beta_{9}\sim Normal(0.16, 0.09)$*,*  $\beta_{10}\sim Normal(-0.17, 0.09)$*,*  $\beta_{11}\sim Normal(0.05, 0.09)$*,*  $\beta_{12}\sim Normal(0.05, 0.12)$*,*  $\beta_{13}\sim Normal(0.07, 0.12)$*.* | priors were  $\beta_{0}\sim Normal(0.23, 0.24)$*,*  $\beta_{1}\sim Normal(0.1, 0.07)$*,*  $\beta_{2}\sim Normal\left( -0.26, 0.09 \right)$*,*  $\beta_{3}\sim Normal(0.21, 0.06)$*,*  $\beta_{4}\sim Normal(0.07, 0.06)$*,*  $\beta_{5}\sim Normal(-0.1, 0.07)$*,*  $\beta_{6}\sim Normal(-0.003, 0.004)$*,*  $\beta_{7}\sim Normal(-0.14, 0.08)$*,*  $\beta_{8}\sim Normal(-0.06, 0.09)$*,*  $\beta_{9}\sim Normal(0.11, 0.09)$*,*  $\beta_{10}\sim Normal(-0.09, 0.09)$*,*  $\beta_{11}\sim Normal(0.003, 0.091)$*,*  $\beta_{12}\sim Normal(0.13, 0.11)$*,*  $\beta_{13}\sim Normal(0.06, 0.12)$*.* |

(priors were taken from E1 3-way interaction model posterior estimates)

### E3: Exploratory model: overall political position

| $y_{ij}=$ | $\beta_{0}*1+$  $\beta_{1}*{Political Position}_{ij}$  $\beta_{2}*\mathrm{Context}_{ij}+$  $\beta_{3}*{Positive General AI Attitude}_{ij}+$  $\beta_{4}*{Negative General AI Attitude}_{ij}+$  $\beta_{5}*{Age}_{ij}+$  $\beta_{6}*{Political Position}_{ij}*\mathrm{Context}_{ij}+$  $\left( 1\vert topic \right)+\left( 1\vert scenario \right)+\left( 1 \vert ID \right)+$  $\varepsilon_{ij}$ |
| --- | --- |

where

$$y_{ij}=\left[ \begin{matrix} {Willingness to Act on AI}_{ij} \\ {Trust in AI}_{ij} \\ {Procedural Fairness of AI}_{ij} \\ {Distributive Fairness of AI}_{ij} \end{matrix} \right]$$

and for

| $y_{ij}={Willingness to Act on AI}_{ij},$ | $y_{ij}={Trust in AI}_{ij},$ | $y_{ij}=\left[ \begin{matrix} {Procedural Fairness of AI}_{ij} \\ {Distributive Fairness of AI}_{ij} \end{matrix} \right],$ |
| --- | --- | --- |
| priors were  $\beta_{0}\sim Normal(0.06, 0.25)$*,*  $\beta_{1}\sim Normal(-0.16, 0.07)$*,*  $\beta_{2}\sim Normal\left( -0.59, 0.09 \right)$*,*  $\beta_{3}\sim Normal(0.08, 0.05)$*,*  $\beta_{4}\sim Normal(0.07, 0.05)$*,*  $\beta_{5}\sim Normal(0.006, 0.004)$*,*  $\beta_{6}\sim Normal(0.31, 0.08)$*.* | priors were  $\beta_{0}\sim Normal(0.16, 0.24)$*,*  $\beta_{1}\sim Normal(-0.08, 0.07)$*,*  $\beta_{2}\sim Normal(-0.25, 0.09)$*,*  $\beta_{3}\sim Normal(0.16, 0.05)$*,*  $\beta_{4}\sim Normal(0.12, 0.05)$*,*  $\beta_{5}\sim Normal(-0.001, 0.004)$*,*  $\beta_{6}\sim Normal(0.13, 0.08)$*.* | priors were  $\beta_{0}\sim Normal(0.18, 0.24)$*,*  $\beta_{1}\sim Normal(-0.12, 0.07)$*,*  $\beta_{2}\sim Normal(-0.26, 0.09)$*,*  $\beta_{3}\sim Normal(0.18, 0.05)$*,*  $\beta_{4}\sim Normal(0.07, 0.05)$*,*  $\beta_{6}\sim Normal(-0.001, 0.004)$*,*  $\beta_{7}\sim Normal(0.14, 0.07)$*.* |

(priors were taken from E1 full model posterior estimates)

### E3: Exploratory model: 3-way interaction: overall political position

| $y_{ij}=$ | $\beta_{0}*1+$  $\beta_{1}*{Political Position}_{ij}+$  $\beta_{2}*\mathrm{Context}_{ij}+$  $\beta_{3}*{Positive General AI Attitude}_{ij}+$  $\beta_{4}*{Negative General AI Attitude}_{ij}+$  $\beta_{5}*{Age}_{ij}+$  $\beta_{6}*{Political Position}_{ij}*\mathrm{Context}_{ij}+$  $\beta_{7}*{Political Position}_{ij}*{Positive General AI Attitude}_{ij}+$  $\beta_{8}*{Political Position}_{ij}*{Negative General AI Attitude}_{ij}+$  $\beta_{9}*\mathrm{Context}_{ij}*{Positive General AI Attitude}_{ij}+$  $\beta_{10}*\mathrm{Context}_{ij}*{Negative General AI Attitude}_{ij}+$  $\beta_{11}*{Political Position}_{ij}*\mathrm{Context}_{ij}*{Positive General AI Attitude}_{ij}+$  $\beta_{12}*{Political Position}_{ij}*\mathrm{Context}_{ij}*{Negative General AI Attitude}_{ij}+$  $\left( 1 \vert topic \right)+\left( 1\vert scenario \right)+\left( 1 \vert ID \right)+$  $\varepsilon_{ij}$ |
| --- | --- |

where

| $y_{ij}={Willingness to Act on AI}_{ij},$ | $y_{ij}={Trust in AI}_{ij},$ | $y_{ij}=\left[ \begin{matrix} {Procedural Fairness of AI}_{ij} \\ {Distributive Fairness of AI}_{ij} \end{matrix} \right],$ |
| --- | --- | --- |
| priors were  $\beta_{0}\sim Normal(0.07, 0.26)$*,*  $\beta_{1}\sim Normal(-0.16, 0.07)$*,*  $\beta_{2}\sim Normal(-0.59, 0.09)$*,*  $\beta_{3}\sim Normal(0.08, 0.06)$*,*  $\beta_{4}\sim Normal(0.07, 0.06)$*,*  $\beta_{5}\sim Normal(0.006, 0.004)$*,*  $\beta_{6}\sim Normal(0.31, 0.08)$*,*  $\beta_{7}\sim Normal(0.01, 0.09)$*,*  $\beta_{8}\sim Normal(-0.01, 0.09)$*,*  $\beta_{9}\sim Normal(-0.01, 0.1)$*,*  $\beta_{10}\sim Normal(-0.01, 0.1)$*,*  $\beta_{11}\sim Normal(-0.01, 0.12)$*,*  $\beta_{12}\sim Normal(-0.01, 0.12)$*.* | priors were  $\beta_{0}\sim Normal(0.23, 0.23)$*,*  $\beta_{1}\sim Normal(-0.06, 0.07)$*,*  $\beta_{2}\sim Normal\left( -0.25, 0.09 \right)$*,*  $\beta_{3}\sim Normal(0.21, 0.06)$*,*  $\beta_{4}\sim Normal(0.1, 0.06)$*,*  $\beta_{5}\sim Normal(-0.002, 0.004)$*,*  $\beta_{6}\sim Normal(0.12, 0.08)$*,*  $\beta_{7}\sim Normal(0.08, 0.09)$*,*  $\beta_{8}\sim Normal(-0.16, 0.09)$*,*  $\beta_{9}\sim Normal(-0.17, 0.09)$*,*  $\beta_{10}\sim Normal(0.05, 0.09)$*,*  $\beta_{11}\sim Normal(-0.05, 0.12)$*,*  $\beta_{12}\sim Normal(-0.07, 0.12)$*.* | priors were  $\beta_{0}\sim Normal(0.23, 0.24)$*,*  $\beta_{1}\sim Normal(-0.1, 0.07)$*,*  $\beta_{2}\sim Normal\left( -0.26, 0.09 \right)$*,*  $\beta_{3}\sim Normal(0.21, 0.06)$*,*  $\beta_{4}\sim Normal(0.07, 0.06)$*,*  $\beta_{5}\sim Normal(-0.003, 0.004)$*,*  $\beta_{6}\sim Normal(0.14, 0.08)$*,*  $\beta_{7}\sim Normal(0.06, 0.09)$*,*  $\beta_{8}\sim Normal(-0.11, 0.09)$*,*  $\beta_{9}\sim Normal(-0.09, 0.09)$*,*  $\beta_{10}\sim Normal(0.003, 0.091)$*,*  $\beta_{11}\sim Normal(-0.13, 0.11)$*,*  $\beta_{12}\sim Normal(-0.06, 0.12)$*.* |

(priors were taken from E1 3-way interaction model posterior estimates)

# Appendix D: Descriptive Statistics

## D.1 Means, Median, and Range

| Table D1: Descriptive summaries of measured variables in Experiment 1 & 2. | | | | | | | | |
| --- | --- | --- | --- | --- | --- | --- | --- | --- |
|  |  | Experiment 1 | | |  | Experiment 2 | | |
|  |  | Mean (SD) | Median | Range |  | Mean (SD) | Median | Range |
| Political Positions (1 = *Very Left/Liberal*, 7 = *Very Right/Conservative*) | | | | | | | | |
|  | Economic Issues | 3.39 (1.33) | 3 | 6 |  | 3.47 (1.34) | 4 | 6 |
|  | Social Issues | 3.14 (1.39) | 3 | 6 |  | 3.16 (1.32) | 3 | 6 |
|  | Foreign Policy Issues | 3.37 (1.34) | 4 | 6 |  | 3.40 (1.40) | 4 | 6 |
|  | Mean Political Position | 3.30 (1.25) | 3.33 | 5.67 |  | 3.34 (1.25) | 3.33 | 6 |
| General Attitudes Towards AI (5-point Likert scale; higher score indicates positive attitudes) | | | | | | | | |
|  | Positive Subscale | 3.33 (0.60) | 3.33 | 2.75 |  | 3.31 (0.60) | 3.33 | 3.5 |
|  | Negative Subscale | 2.97 (0.65) | 3 | 3.25 |  | 3.04 (0.69) | 3.12 | 3.75 |
| Responses to Scenarios (1 = *Strongly Disagree*, 5 = *Strongly Agree*) | | | | | | | | |
|  | Willingness to Act | 3.94 (0.91) | 4.07 | 4 |  | 3.90 (0.93) | 4.07 | 4 |
|  | Trust | 3.56 (0.86) | 3.63 | 4 |  | 3.44 (0.92) | 3.7 | 4 |
|  | Perceived Fairness | 3.68 (0.92) | 3.95 | 4 |  | 3.56 (0.94) | 3.78 | 4 |
| *Note.* For meaningful interpretations, descriptive statistics are presented in original scales. | | | | | | | | |

| Table D2: Descriptive summaries of measured variables in Experiment 3. | | | | | |
| --- | --- | --- | --- | --- | --- |
|  |  | | Mean (SD) | Median | Range |
| Political Positions (1 = *Very Left/Liberal*, 7 = *Very Right/Conservative*) | | | | | |
|  | Economic Issues | | 3.25 (1.38) | 3 | 6 |
|  | Social Issues | | 3.05 (1.35) | 3 | 6 |
|  | Foreign Policy Issues | | 3.30 (1.35) | 4 | 6 |
|  | Mean Political Position | | 3.20 (1.28) | 3.33 | 6 |
| Issue-Specific Attitudes (higher score indicates positive attitudes) | | | | | |
|  | LGBTQ Rights | | | | |
|  |  | Temperate Scale (1° ~100°) | 72.22 (29.97) | 82 | 100 |
|  |  | Extent of Concern (5-point Likert scale) | 3.40 (1.16) | 4 | 4 |
|  | Environmental Protection | | | | |
|  |  | Temperate Scale (1° ~100°) | 73.55 (22.18) | 78 | 100 |
|  |  | Extent of Concern (5-point Likert scale) | 4.06 (0.88) | 4 | 4 |
| General Attitudes Towards AI (5-point Likert scale; higher score indicates positive attitudes) | | | | | |
|  | Positive Subscale | | 3.23 (0.63) | 3.33 | 3.75 |
|  | Negative Subscale | | 3.03 (0.68) | 3.12 | 3.5 |
| Responses to Scenarios (1 = *Strongly Disagree*, 5 = *Strongly Agree*) | | | | | |
|  | Willingness to Act | | 3.15 (1.16) | 3.04 | 4 |
|  | Trust | | 3.39 (0.94) | 3.49 | 4 |
|  | Procedural Fairness | | 3.74 (0.92) | 3.99 | 4 |
|  | Distributive Fairness | | 3.36 (1.05) | 3.53 | 4 |
| *Note.* For meaningful interpretations, descriptive statistics are presented in original scales. | | | | | |

## D.2 Descriptive Boxplots


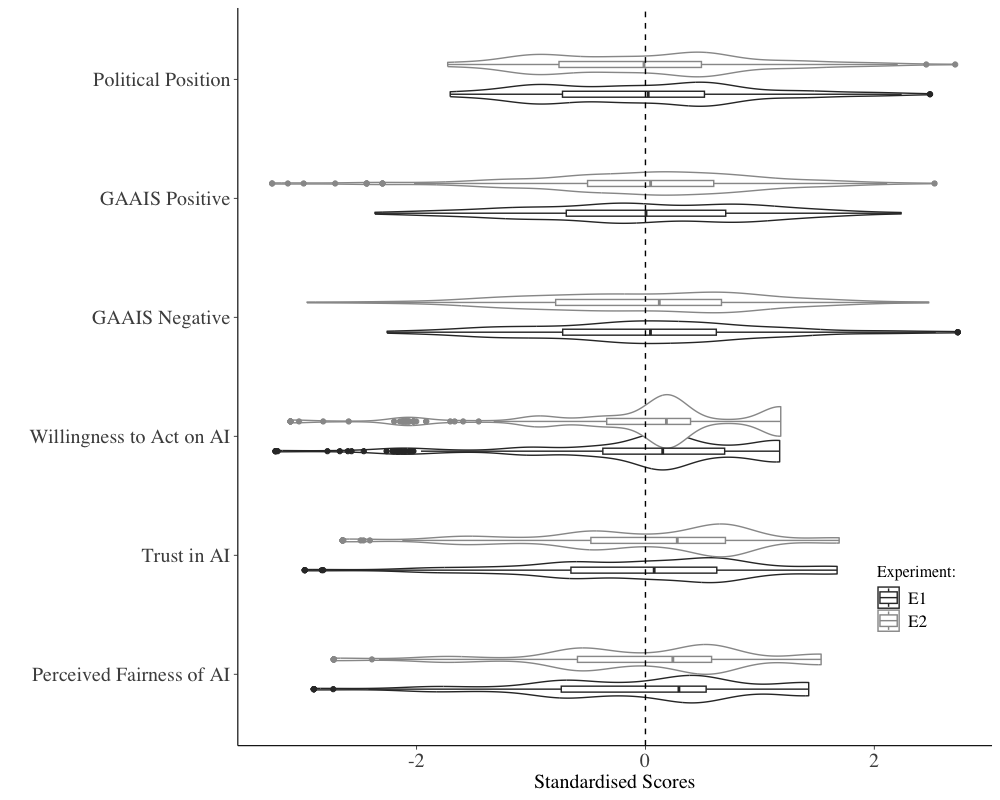


Figure D1: Descriptive boxplots of standardised measured variables in E1 & E2.


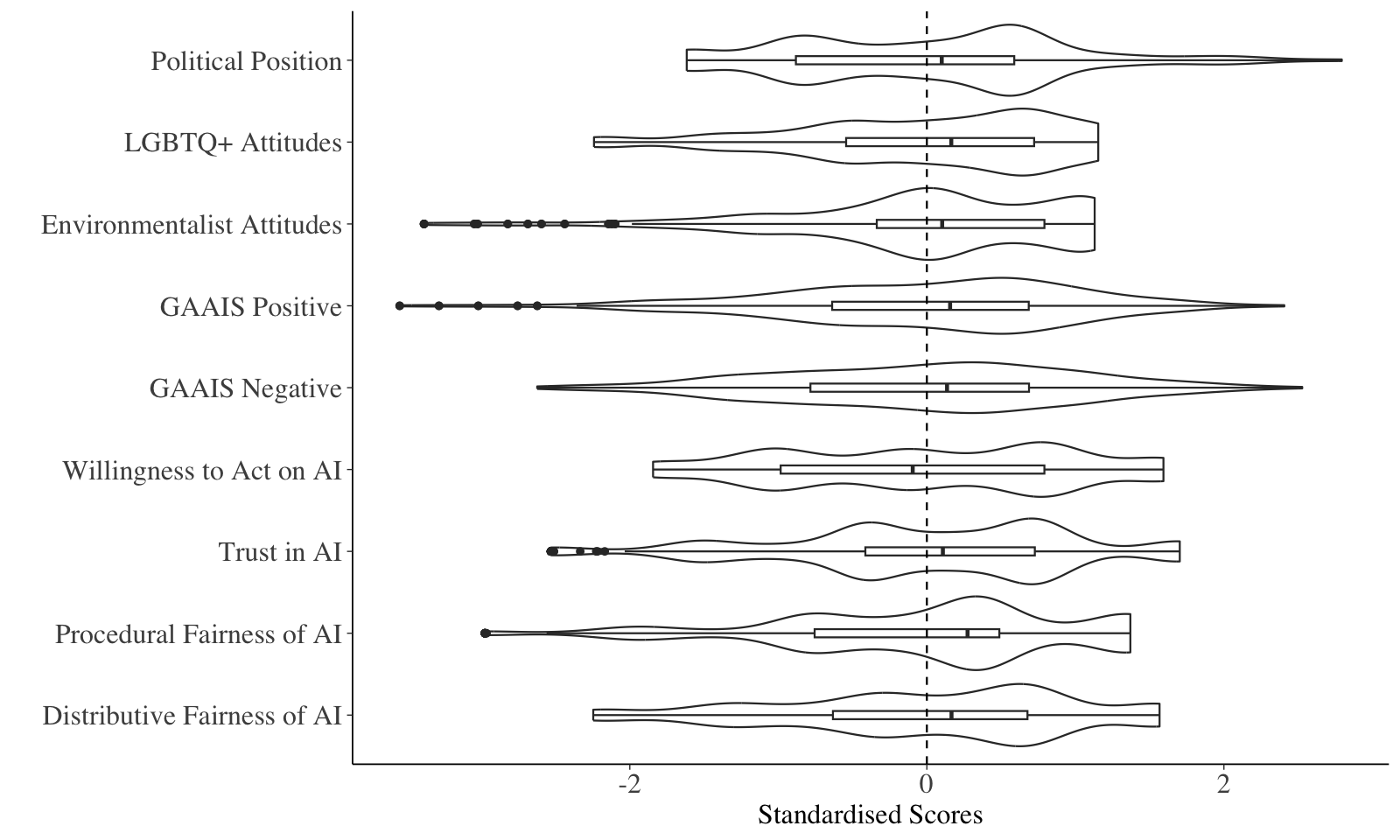


Figure D2: Descriptive boxplots of standardised measured variables in E3.

## D.3 Bayesian Pearson’s Correlation Tables

| Table D3: Bayesian Pearson’s zero-order correlations and their 95% *HDI*s between main variables  in Experiment 1 (E1; lower diagonal) and Experiment 2 (E2; upper diagonal). | | | | | | |
| --- | --- | --- | --- | --- | --- | --- |
| E2  E1 | Political Positions | GAAIS Positive | GAAIS Negative | Willingness to Act | Trust | Perceived Fairness |
| Political Positions | 1 | -0.13**  [-0.24, -0.02] | -0.13**  [-0.25, -0.02] | 0.03  [-0.09, 0.14] | 0  [-0.1, 0.12] | -0.06  [-0.17, 0.06] |
| GAAIS Positive | -0.07  [-0.16, 0.03] | 1 | 0.5***  [0.41, 0.58] | 0.07  [-0.04, 0.18] | -0.01  [-0.12, 0.11] | 0.02  [-0.09, 0.13] |
| GAAIS Negative | 0.05  [-0.04, 0.14] | 0.51***  [0.43, 0.57] | 1 | 0  [-0.11, 0.11] | -0.02  [-0.13, 0.09] | 0.02  [-0.09, 0.14] |
| Willingness to Act | 0  [-0.1, 0.1] | 0.07  [-0.03, 0.17] | 0.06  [-0.03, 0.16] | 1 | 0.35***  [0.26, 0.45] | 0.36***  [0.26, 0.46] |
| Trust | -0.02  [-0.12, 0.07] | 0.19***  [0.1, 0.29] | 0.15***  [0.05, 0.24] | 0.3***  [0.21, 0.39] | 1 | 0.63***  [0.56, 0.69] |
| Perceived Fairness | -0.06  [-0.16, 0.03] | 0.21***  [0.11, 0.3] | 0.11*  [0.01, 0.2] | 0.36***  [0.27, 0.44] | 0.61***  [0.55, 0.67] | 1 |
| *Note*. Probability of direction (pd) represents the portion of the posterior distribution in the same direction of effect as the median (Makowski et al., 2019); *** pd > 99.95%, ** pd > 99.5%, * pd > 97.5%. GAAIS Negative values are reverse-coded. | | | | | | |

| Table D4: Bayesian Pearson’s zero-order correlations and their 95% *HDI*s between main variables in Experiment 3. | | | | | | | | |
| --- | --- | --- | --- | --- | --- | --- | --- | --- |
|  | Political Positions | LGBTQ+ Rights | Environment-al Protection | GAAIS Positive | GAAIS Negative | Willingness to Act | Trust | Procedural Fairness |
| LGBTQ+  Rights | -0.46***  [-0.53, -0.41] |  |  |  |  |  |  |  |
| Environment-al Protection | -0.34***  [-0.41, -0.26] | 0.49***  [0.43, 0.55] |  |  |  |  |  |  |
| GAAIS Positive | -0.12**  [-0.19, -0.04] | 0.22***  [0.14, 0.29] | 0.22***  [0.15, 0.30] |  |  |  |  |  |
| GAAIS Negative | 0.08*  [0, 0.15] | 0.08*  [0, 0.16] | -0.04  [-0.12, 0.04] | 0.36***  [0.29, 0.43] |  |  |  |  |
| Willingness to Act | -0.12***  [-0.20, -0.04] | 0.19***  [0.12, 0.27] | 0.17***  [0.09, 0.24] | 0.11**  [0.03, 0.18] | 0.04  [-0.04, 0.12] |  |  |  |
| Trust | -0.04  [-0.12, 0.04] | 0.16***  [0.08, 0.24] | 0.27***  [0.20, 0.34] | 0.41***  [0.34, 0.47] | 0.25***  [0.17, 0.32] | 0.46***  [0.39, 0.52] |  |  |
| Procedural Fairness | -0.06  [-0.14, 0.02] | 0.18***  [0.11, 0.26] | 0.25***  [0.17, 0.32] | 0.28***  [0.21, 0.36] | 0.16***  [0.07, 0.23] | 0.48***  [0.42, 0.54] | 0.61***  [0.56, 0.66] |  |
| Distributive Fairness | -0.01  [-0.09, 0.07] | 0.17***  [0.09, 0.24] | 0.25***  [0.18, 0.33] | 0.39***  [0.32, 0.45] | 0.23***  [0.16, 0.31] | 0.35***  [0.28, 0.42] | 0.58***  [0.53, 0.63] | 0.55***  [0.49, 0.61] |
| *Note*. Probability of direction (pd) represents the portion of the posterior distribution in the same direction of effect as the median (Makowski et al., 2019); *** pd > 99.95%, ** pd > 99.5%, * pd > 97.5%. GAAIS Negative values are reverse-coded. | | | | | | | | |

## D.4 Bayesian Pearson’s Correlation Matrixes


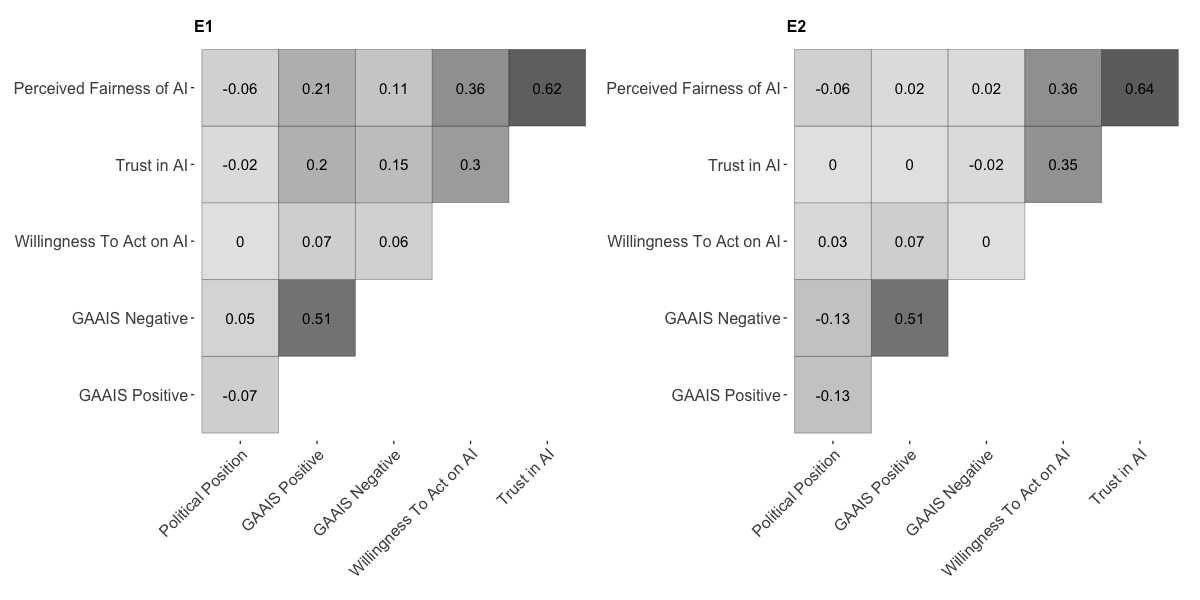


Figure D3: Bayesian Pearson’s zero-order correlations between main variables in E1 & E2, with darker squares indicating stronger correlations.


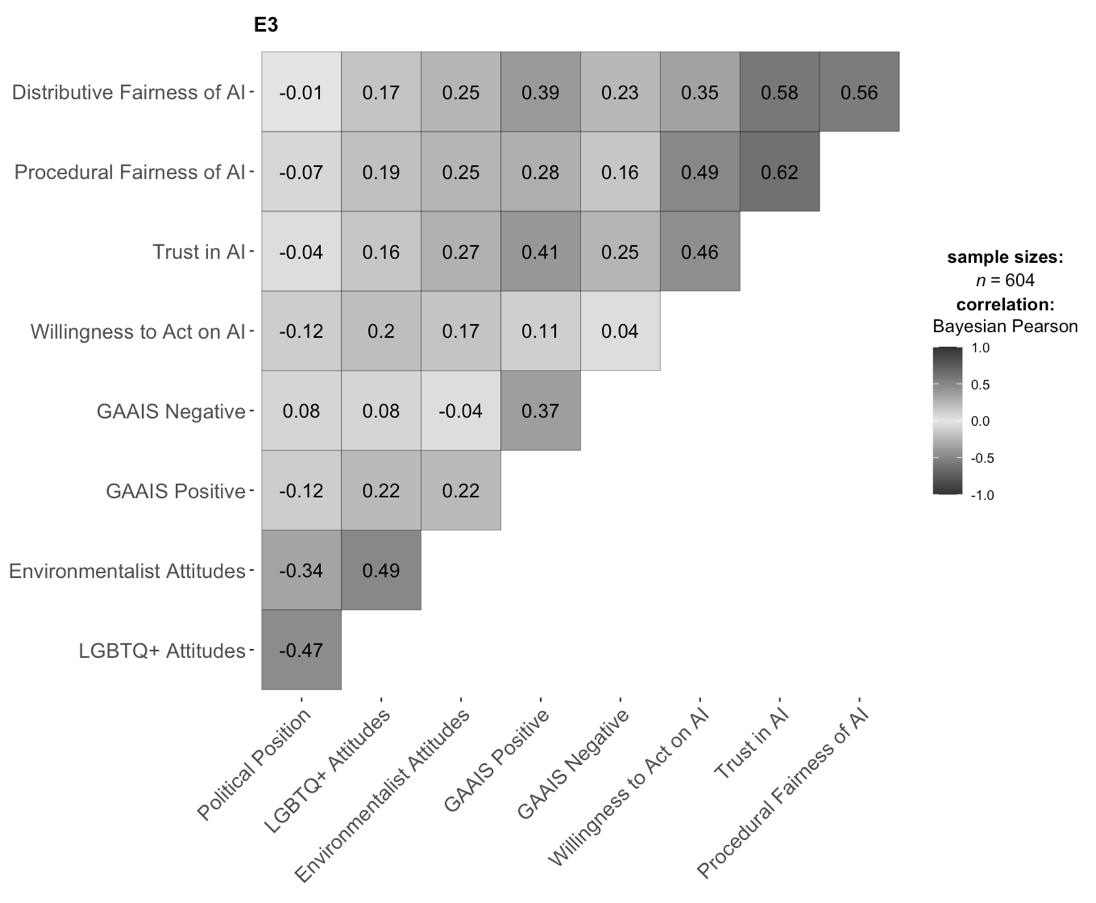


Figure D4: Bayesian Pearson’s zero-order correlations between main variables in E3, with darker squares indicating stronger correlations.

## D.5 Observed Data Plots


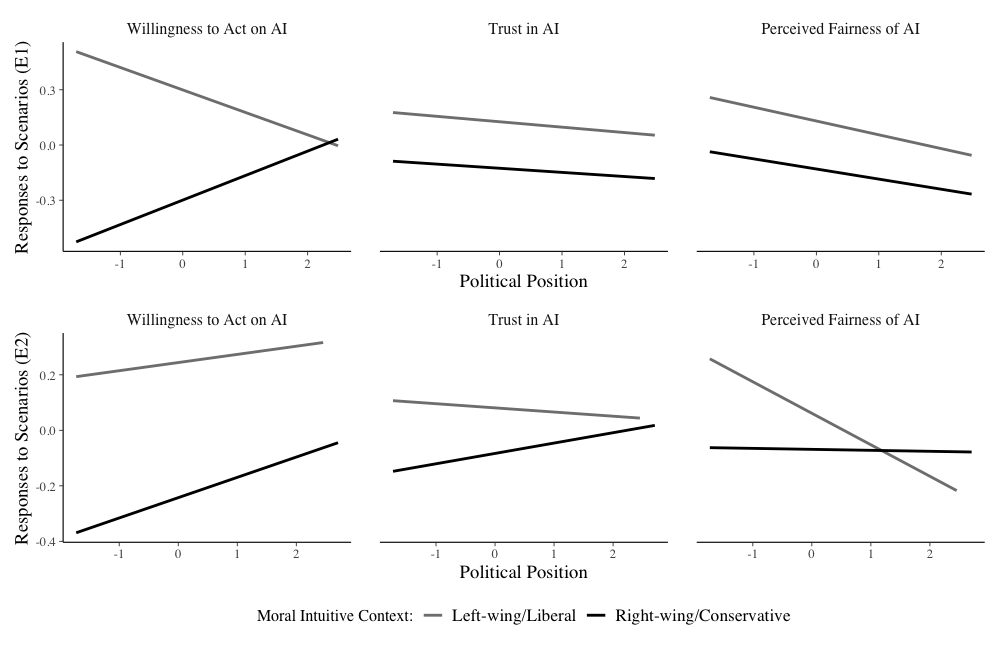


Figure D5: Observed interactions between participant political position and moral intuitive context of AI verdicts for Willingness to Act on AI verdicts, Trust in AI, and Perceived Fairness of AI in E1 & E2. Higher scores on political position correspond to increasing conservatism.


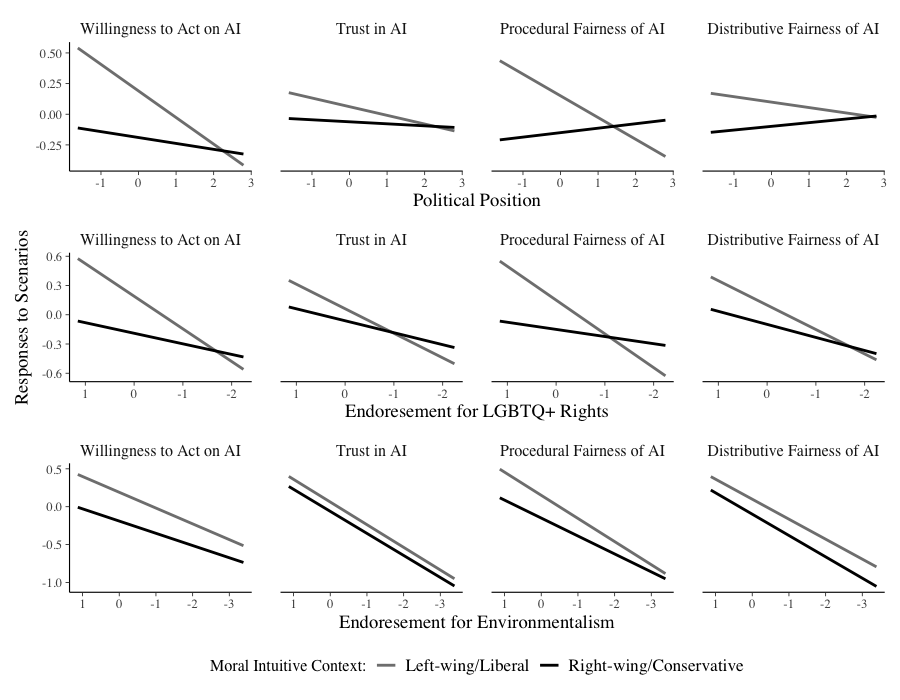


Figure D6: Observed interactions between participant political position and moral intuitive context of AI verdicts for Willingness to Act on AI verdicts, Trust in AI, perception of Procedural Fairness and Distributive Fairness of AI in E3. Higher scores on political position correspond to increasing conservatism, and higher scores on LGBTQ rights and environmental protection indicate greater endorsement.

# Appendix E: Model Regression Output & Interaction Effects

## E.1 Summaries of Bayesian Regression Results

| Table E1: Summaries of Bayesian regression results in Experiment 1 & 2. | | | | | | |
| --- | --- | --- | --- | --- | --- | --- |
| **Experiment 1** | Willingness to Act | | Trust | | Fairness Perception | |
|  | Mean [95% *HDI*] | *SD* | Mean [95% *HDI*] | *SD* | Mean [95% *HDI*] | *SD* |
| Intercept | 0.06 [-0.44, 0.56] | 0.25 | 0.16 [-0.31, 0.63] | 0.24 | 0.18 [-0.29, 0.65] | 0.24 |
| Political Position | **-0.16 [-0.29, -0.03]** | 0.07 | -0.08 [-0.21, 0.05] | 0.07 | -0.12 [-0.25, 0.02] | 0.07 |
| Context | **-0.59 [-0.77, -0.41]** | 0.09 | **-0.25 [-0.43, -0.07]** | 0.09 | **-0.26 [-0.43, -0.08]** | 0.09 |
| GAAIS Positive | 0.08 [-0.01, 0.18] | 0.05 | **0.16 [0.06, 0.25]** | 0.05 | **0.18 [0.09, 0.28]** | 0.05 |
| GAAIS Negative | 0.07 [-0.02, 0.16] | 0.05 | **0.12 [0.02, 0.21]** | 0.05 | 0.07 [-0.03, 0.17] | 0.05 |
| Age | 0.01 [0, 0.01] | 0 | 0 [-0.01, 0.01] | 0 | 0 [-0.01, 0.01] | 0 |
| Political Position  x Context | **0.31 [0.15, 0.47]** | 0.08 | 0.13 [-0.03, 0.28] | 0.08 | 0.14 [-0.01, 0.28] | 0.07 |
| **Experiment 2** | Willingness to Act | | Trust | | Fairness Perception | |
|  | Mean [95% *HDI*] | *SD* | Mean [95% *HDI*] | *SD* | Mean [95% *HDI*] | *SD* |
| Intercept | 0.35 [-0.19, 0.89] | 0.27 | 0.08 [-0.44, 0.59] | 0.26 | 0 [-0.51, 0.51] | 0.26 |
| Political Position | -0.01 [-0.16, 0.15] | 0.08 | -0.07 [-0.23, 0.09] | 0.08 | -0.16 [-0.32, 0] | 0.08 |
| Context | **-0.46 [-0.69, -0.24]** | 0.11 | -0.14 [-0.37, 0.09] | 0.12 | -0.11 [-0.33, 0.13] | 0.12 |
| GAAIS Positive | 0.1 [-0.01, 0.2] | 0.05 | 0.06 [-0.04, 0.16] | 0.05 | 0.07 [-0.03, 0.18] | 0.05 |
| GAAIS Negative | 0.02 [-0.08, 0.13] | 0.05 | 0.05 [-0.05, 0.15] | 0.05 | 0.08 [-0.03, 0.18] | 0.05 |
| Age | 0 [-0.01, 0.01] | 0 | 0 [-0.01, 0.01] | 0 | 0 [-0.01, 0.01] | 0 |
| Political Position  x Context | **0.19 [0, 0.37]** | 0.09 | **0.2 [0.02, 0.38]** | 0.09 | **0.24 [0.06, 0.41]** | 0.09 |
| *Note*. Model converged successfully with split $\hat{R}$ = 1 for all estimated parameters. Context is a binary variable with liberal/left-wing direction as the reference level. GAAIS Negative values are reverse-coded. Bold emphasises 0 ∉ 95% *HDI*. | | | | | | |

| Table E2: Summaries of Bayesian regression results in Experiment 3. | | | | | | | | |
| --- | --- | --- | --- | --- | --- | --- | --- | --- |
| **(a)**  **LGBTQ+ Rights** | Willingness to Act | | Trust | | Procedural Fairness | | Distributive Fairness | |
|  | Mean  [95% *HDI*] | *SD* | Mean  [95% *HDI*] | *SD* | Mean  [95% *HDI*] | *SD* | Mean  [95% *HDI*] | *SD* |
| Intercept | 0.2  [-0.26,0.64] | 0.23 | 0.13  [-0.21,0.54] | 0.18 | 0.3  [-0.04,0.7] | 0.18 | 0.11  [-0.23,0.52] | 0.18 |
| LGBTQ+ Attitudes | **0.21**  **[0.11,0.31]** | 0.05 | **0.11**  **[0.02,0.21]** | 0.05 | **0.18**  **[0.08,0.27]** | 0.05 | **0.11**  **[0.01,0.2]** | 0.05 |
| Context | **-0.5**  **[-0.63,-0.37]** | 0.07 | **-0.22**  **[-0.35,-0.1]** | 0.07 | **-0.29**  **[-0.42,-0.17]** | 0.06 | **-0.21**  **[-0.34,-0.09]** | 0.06 |
| GAAIS Positive | 0.04  [-0.03,0.11] | 0.04 | **0.26**  **[0.19,0.33]** | 0.04 | **0.16**  **[0.09,0.23]** | 0.04 | **0.25**  **[0.18,0.32]** | 0.04 |
| GAAIS Negative | 0.05  [-0.02,0.12] | 0.04 | **0.12**  **[0.05,0.18]** | 0.04 | **0.09**  **[0.02,0.16]** | 0.03 | **0.1**  **[0.04,0.17]** | 0.03 |
| Political Position | **-0.15**  **[-0.24,-0.07]** | 0.04 | -0.04  [-0.13,0.04] | 0.04 | -0.08  [-0.17,0.01] | 0.04 | -0.07  [-0.15,0.02] | 0.04 |
| Age | 0  [0,0.01] | 0 | 0  [0,0.01] | 0 | 0  [-0.01,0] | 0 | 0  [0,0.01] | 0 |
| LGBTQ+ Attitudes  x Context | **-0.22**  **[-0.34,-0.1]** | 0.06 | **-0.12**  **[-0.24,0]** | 0.06 | **-0.18**  **[-0.29,-0.07]** | 0.06 | **-0.13**  **[-0.24,-0.02]** | 0.06 |
| **(b)**  **Environmental Concerns** | Willingness to Act | | Trust | | Procedural Fairness | | Distributive Fairness | |
|  | Mean  [95% *HDI*] | *SD* | Mean  [95% *HDI*] | *SD* | Mean  [95% *HDI*] | *SD* | Mean  [95% *HDI*] | *SD* |
| Intercept | 0.06  [-0.3,0.5] | 0.2 | 0.18  [-0.15,0.58] | 0.18 | 0.23  [-0.15,0.68] | 0.21 | 0.06  [-0.29,0.5] | 0.2 |
| Environmentalist Attitudes | **0.15**  **[0.05,0.24]** | 0.05 | **0.12**  **[0.04,0.21]** | 0.04 | **0.19**  **[0.1,0.28]** | 0.05 | **0.19**  **[0.1,0.28]** | 0.05 |
| Context | **-0.51**  **[-0.64,-0.38]** | 0.07 | **-0.19**  **[-0.3,-0.07]** | 0.06 | **-0.34**  **[-0.47,-0.22]** | 0.06 | **-0.27**  **[-0.4,-0.15]** | 0.06 |
| GAAIS Positive | 0.05  [-0.02,0.13] | 0.04 | **0.23**  **[0.17,0.3]** | 0.03 | **0.17**  **[0.1,0.24]** | 0.04 | **0.24**  **[0.17,0.31]** | 0.04 |
| GAAIS Negative | 0.04  [-0.04,0.11] | 0.04 | **0.15**  **[0.09,0.22]** | 0.03 | 0.06  [0,0.13] | 0.03 | **0.1**  **[0.04,0.17]** | 0.03 |
| Political Position | **-0.15**  **[-0.24,-0.06]** | 0.04 | -0.06  [-0.14,0.02] | 0.04 | -0.07  [-0.16,0.01] | 0.04 | -0.01  [-0.1,0.07] | 0.04 |
| Age | 0  [0,0.01] | 0 | 0  [-0.01,0] | 0 | 0  [-0.01,0.01] | 0 | 0  [0,0.01] | 0 |
| Env. Attitudes  x Context | **-0.29**  **[-0.41,-0.17]** | 0.06 | -0.04[-0.14,0.07] | 0.06 | **-0.16**  **[-0.26,-0.05]** | 0.05 | **-0.12**  **[-0.22,-0.01]** | 0.06 |
| **(c)**  **Political Position (exploratory)** | Willingness to Act | | Trust | | Procedural Fairness | | Distributive Fairness | |
|  | Mean  [95% *HDI*] | *SD* | Mean  [95% *HDI*] | *SD* | Mean  [95% *HDI*] | *SD* | Mean  [95% *HDI*] | *SD* |
| Intercept | 0.15  [-0.32,0.63] | 0.24 | 0.14  [-0.23,0.58] | 0.2 | 0.29  [-0.1,0.75] | 0.21 | 0.08  [-0.3,0.53] | 0.21 |
| Political Position | **-0.2**  **[-0.29,-0.12]** | 0.04 | -0.07  [-0.14,0.01] | 0.04 | **-0.14**  **[-0.22,-0.07]** | 0.04 | -0.07  [-0.15,0.01] | 0.04 |
| Context | **-0.45**  **[-0.54,-0.36]** | 0.05 | **-0.18**  **[-0.28,-0.09]** | 0.05 | **-0.34**  **[-0.43,-0.24]** | 0.05 | **-0.23**  **[-0.32,-0.15]** | 0.04 |
| GAAIS Positive | 0.06  [-0.01,0.12] | 0.03 | **0.28**  **[0.22,0.34]** | 0.03 | **0.19**  **[0.13,0.25]** | 0.03 | **0.27**  **[0.21,0.34]** | 0.03 |
| GAAIS Negative | 0.04  [-0.02,0.11] | 0.03 | **0.13**  **[0.07,0.19]** | 0.03 | **0.08**  **[0.02,0.14]** | 0.03 | **0.11**  **[0.04,0.17]** | 0.03 |
| Age | 0  [0,0.01] | 0 | 0  [0,0.01] | 0 | 0  [-0.01,0] | 0 | 0  [0,0.01] | 0 |
| Political Position  x Context | **0.18**  **[0.1,0.27]** | 0.04 | **0.1**  **[0.01,0.19]** | 0.05 | **0.21**  **[0.13,0.29]** | 0.04 | **0.11**  **[0.03,0.19]** | 0.04 |
| *Note*. Model converged successfully with split $\hat{R}$ = 1 for all estimated parameters. Context is a binary variable with liberal/left-wing direction as the reference level. GAAIS Negative values are reverse-coded. Bold emphasises 0 ∉ 95% *HDI*. | | | | | | | | |

## E.2 Dot-and-Whisker Plots of Posterior Estimates


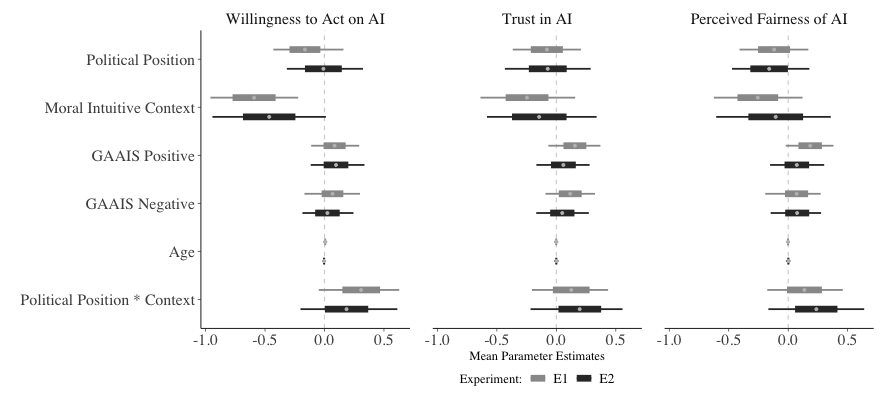


Figure E1: Parameter estimates for Willingness to Act on AI verdicts, Trust in AI, and Perceived Fairness of AI in E1 & E2, with boxes indicating 95% *HDI*s and whiskers indicating 100% *HDI*s. Higher standardised scores on political position correspond to increasing conservatism. Context is a binary variable with liberal/left-wing direction as the reference level.


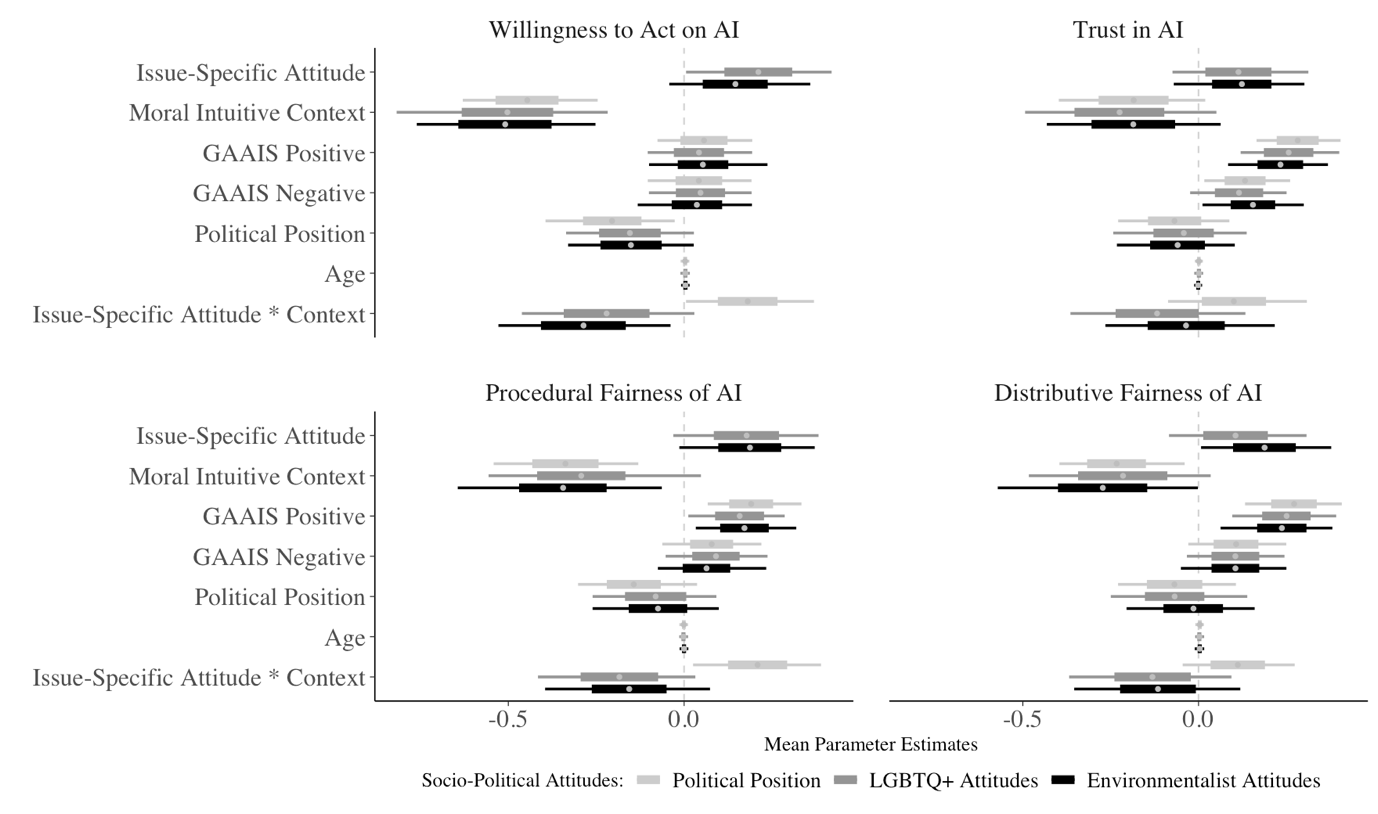


Figure E2: Parameter estimates for Willingness to Act on AI verdicts, Trust in AI and perception of Procedural Fairness and Distributive Fairness of AI in E3, with boxes indicating 95% *HDI*s and whiskers indicating 100% *HDI*s. Higher standardised scores on political position correspond to increasing conservatism. Context is a binary variable with liberal/left-wing direction as the reference level. Neither issue-specific attitudes was used as a predictor when modelling effects of belief alignment between general political position (Conservatism) and moral intuitive context of AI detection.

## E.2 Model Interaction Plots


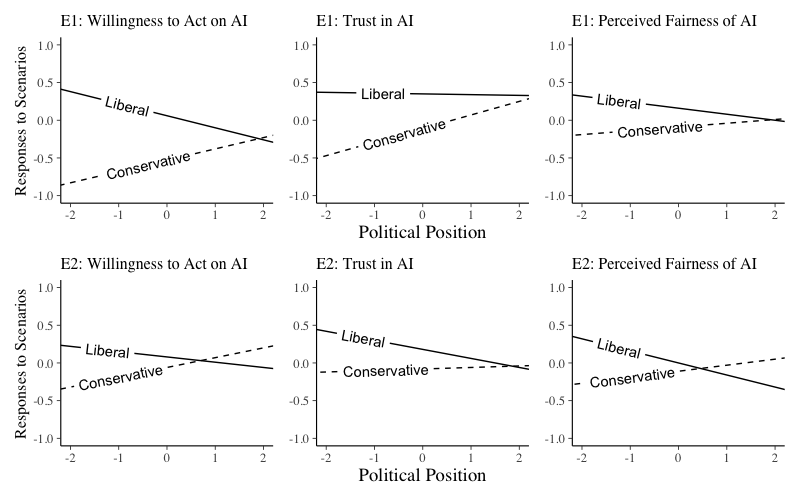


Figure E3: Interactions between participant political position and moral intuitive context of AI verdicts for Willingness to Act on AI verdicts, Trust in AI, and Perceived Fairness of AI in E1 & E2. Higher scores on political position correspond to increasing conservatism.


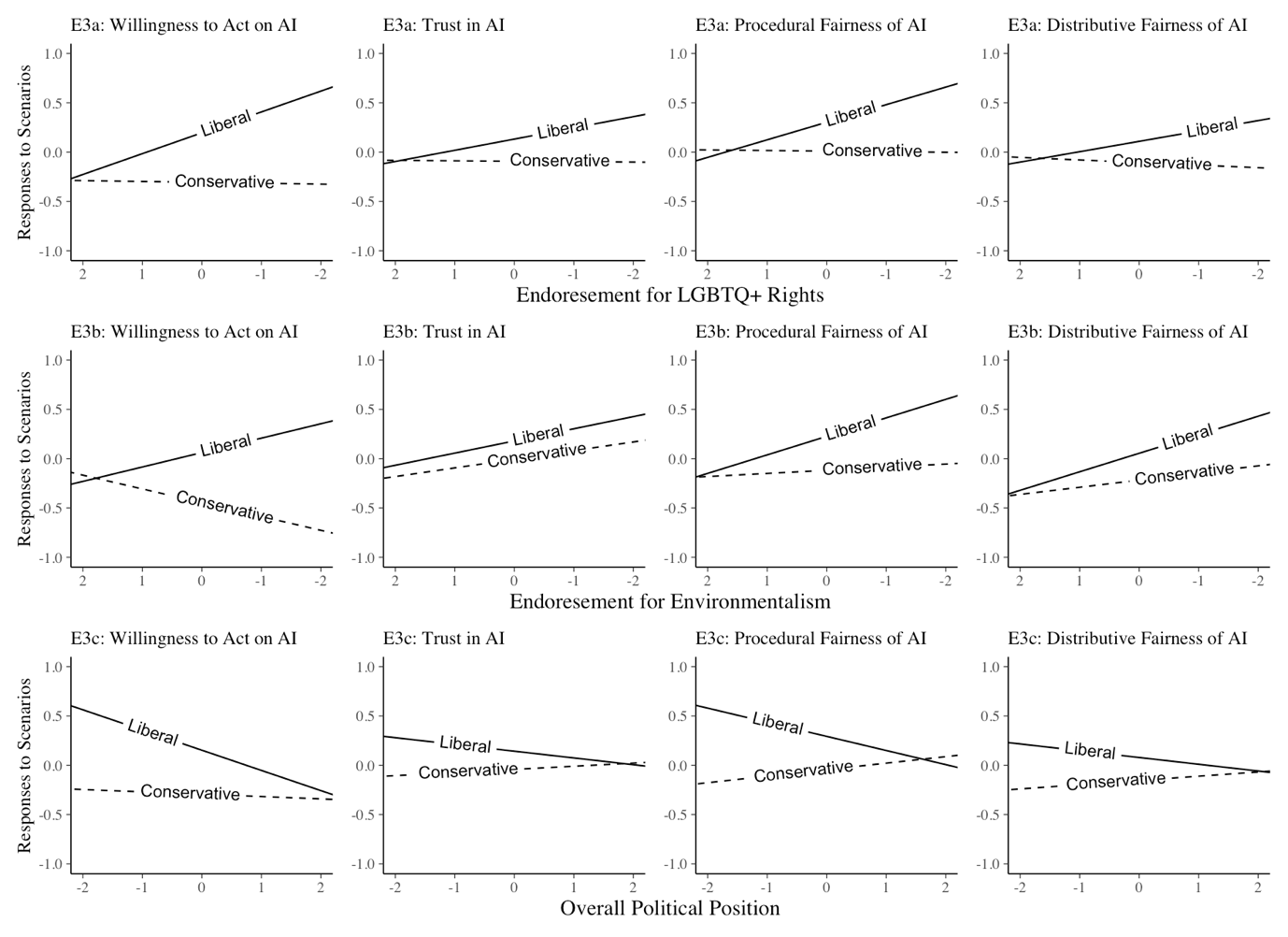


Figure E4: Interactions between participant political position and moral intuitive context of AI verdicts for Willingness to Act on AI verdicts, Trust in AI, perception of Procedural Fairness and Distributive Fairness of AI in E3. Higher scores on political position correspond to increasing conservatism, and higher scores on LGBTQ rights and environmental protection indicate greater endorsement.

## E.4 Summaries of Bayesian Regression Results – Exploratory Models & Model Comparison

| Table E4: Bayesian regression results for exploratory three-way interaction models in Experiment 1 & 2. | | | | | | |
| --- | --- | --- | --- | --- | --- | --- |
| **Experiment 1** | Willingness to Act | | Trust | | Fairness Perception | |
|  | Mean [95% *HDI*] | *SD* | Mean [95% *HDI*] | *SD* | Mean [95% *HDI*] | *SD* |
| Intercept | 0.07 [-0.43, 0.58] | 0.25 | 0.23 [-0.24, 0.69] | 0.23 | 0.23 [-0.24, 0.71] | 0.24 |
| Political Position | **-0.16 [-0.3, -0.03]** | 0.07 | -0.06 [-0.2, 0.07] | 0.07 | -0.1 [-0.24, 0.03] | 0.07 |
| Context | **-0.59 [-0.77, -0.41]** | 0.09 | **-0.25 [-0.43, -0.07]** | 0.09 | **-0.26 [-0.43, -0.09]** | 0.09 |
| GAAIS Positive | 0.08 [-0.03, 0.2] | 0.06 | **0.21 [0.09, 0.33]** | 0.06 | **0.21 [0.1, 0.32]** | 0.06 |
| GAAIS Negative | 0.07 [-0.04, 0.19] | 0.06 | 0.1 [-0.02, 0.21] | 0.06 | 0.07 [-0.04, 0.18] | 0.06 |
| Age | 0.01 [0, 0.01] | 0 | 0 [-0.01, 0.01] | 0 | 0 [-0.01, 0.01] | 0 |
| Political Position  x Context | **0.31 [0.15, 0.47]** | 0.08 | 0.12 [-0.04, 0.27] | 0.08 | 0.14 [-0.01, 0.28] | 0.08 |
| Political Position  x GAAIS Positive | 0.01 [-0.16, 0.18] | 0.09 | 0.08 [-0.09, 0.25] | 0.09 | 0.06 [-0.11, 0.23] | 0.09 |
| Political Position  x GAAIS Negative | -0.01 [-0.18, 0.17] | 0.09 | -0.16 [-0.34, 0.02] | 0.09 | -0.11 [-0.29, 0.07] | 0.09 |
| Context  x GAAIS Positive | -0.01 [-0.21, 0.18] | 0.1 | -0.17 [-0.35, 0.02] | 0.1 | -0.09 [-0.27, 0.09] | 0.09 |
| Context  x GAAIS Negative | -0.01 [-0.2, 0.18] | 0.1 | 0.05 [-0.14, 0.23] | 0.09 | 0 [-0.18, 0.18] | 0.09 |
| Political Position  x Context  x GAAIS Positive | -0.01 [-0.24, 0.23] | 0.12 | -0.05 [-0.28, 0.18] | 0.12 | -0.13 [-0.35, 0.09] | 0.11 |
| Political Position  x Context  x GAAIS Negative | -0.01 [-0.26, 0.23] | 0.12 | -0.07 [-0.31, 0.17] | 0.12 | -0.06 [-0.29, 0.17] | 0.12 |
| **Experiment 2** | Willingness to Act | | Trust | | Fairness Perception | |
|  | Mean [95% *HDI*] | *SD* | Mean [95% *HDI*] | *SD* | Mean [95% *HDI*] | *SD* |
| Intercept | 0.36 [-0.17, 0.89] | 0.27 | 0.11 [-0.4, 0.62] | 0.26 | 0.02 [-0.49, 0.53] | 0.26 |
| Political Position | 0 [-0.16, 0.15] | 0.08 | -0.07 [-0.23, 0.09] | 0.08 | -0.15 [-0.31, 0.01] | 0.08 |
| Context | **-0.46 [-0.69, -0.24]** | 0.11 | -0.14 [-0.37, 0.1] | 0.12 | -0.09 [-0.32, 0.14] | 0.12 |
| GAAIS Positive | 0.08 [-0.05, 0.22] | 0.07 | 0.05 [-0.09, 0.18] | 0.07 | 0.08 [-0.05, 0.22] | 0.07 |
| GAAIS Negative | 0.05 [-0.08, 0.18] | 0.07 | 0.02 [-0.11, 0.14] | 0.07 | 0.06 [-0.07, 0.19] | 0.07 |
| Age | 0 [-0.01, 0.01] | 0 | 0 [-0.01, 0.01] | 0 | 0 [-0.01, 0.01] | 0 |
| Political Position  x Context | **0.19 [0.01, 0.37]** | 0.09 | **0.2 [0.02, 0.39]** | 0.09 | **0.25 [0.07, 0.43]** | 0.09 |
| Political Position  x GAAIS Positive | 0.01 [-0.22, 0.23] | 0.12 | -0.08 [-0.32, 0.17] | 0.12 | -0.04 [-0.27, 0.2] | 0.12 |
| Political Position  x GAAIS Negative | 0.08 [-0.12, 0.28] | 0.1 | 0.03 [-0.18, 0.24] | 0.11 | 0.08 [-0.13, 0.29] | 0.11 |
| Context  x GAAIS Positive | 0.03 [-0.19, 0.25] | 0.11 | -0.01 [-0.23, 0.21] | 0.11 | -0.07 [-0.29, 0.15] | 0.11 |
| Context  x GAAIS Negative | -0.06 [-0.28, 0.16] | 0.11 | 0.09 [-0.13, 0.32] | 0.11 | 0.05 [-0.17, 0.27] | 0.11 |
| Political Position  x Context  x GAAIS Positive | 0.01 [-0.27, 0.3] | 0.15 | 0.14 [-0.16, 0.43] | 0.15 | 0.18 [-0.12, 0.48] | 0.15 |
| Political Position  x Context  x GAAIS Negative | 0.02 [-0.24, 0.29] | 0.13 | -0.04 [-0.32, 0.23] | 0.14 | -0.06 [-0.34, 0.21] | 0.14 |
| *Note*. Model converged successfully with split $\hat{R}$ = 1 for all estimated parameters. Context is a binary variable with liberal/left-wing direction as the reference level. GAAIS Negative values are reverse-coded. Bold emphasises 0 ∉ 95% *HDI*. | | | | | | |

| Table E4: Bayesian regression results for exploratory three-way interaction models in Experiment 3. | | | | | | | | |
| --- | --- | --- | --- | --- | --- | --- | --- | --- |
| **(a)**  **LGBTQ+ Rights (exploratory)** | Willingness to Act | | Trust | | Procedural Fairness | | Distributive Fairness | |
|  | Mean  [95% *HDI*] | *SD* | Mean  [95% *HDI*] | *SD* | Mean  [95% *HDI*] | *SD* | Mean  [95% *HDI*] | *SD* |
| Intercept | 0.24  [-0.23,0.71] | 0.24 | 0.2  [-0.14,0.63] | 0.19 | **0.38**  **[0.03,0.8]** | 0.19 | 0.18  [-0.16,0.61] | 0.19 |
| LGBTQ+ Attitudes | **0.21**  **[0.11,0.3]** | 0.05 | **0.1**  **[0,0.19]** | 0.05 | **0.17**  **[0.07,0.26]** | 0.05 | 0.09  [0,0.19] | 0.05 |
| Context | **-0.51**  **[-0.64,-0.38]** | 0.07 | **-0.22**  **[-0.35,-0.1]** | 0.07 | **-0.29**  **[-0.41,-0.16]** | 0.07 | **-0.22**  **[-0.34,-0.09]** | 0.06 |
| GAAIS Positive | 0.03  [-0.06,0.12] | 0.04 | **0.33**  **[0.24,0.41]** | 0.04 | **0.19**  **[0.11,0.28]** | 0.04 | **0.27**  **[0.19,0.36]** | 0.04 |
| GAAIS Negative | 0.06  [-0.03,0.14] | 0.04 | **0.1**  **[0.02,0.18]** | 0.04 | 0.08  [0,0.16] | 0.04 | **0.1**  **[0.02,0.18]** | 0.04 |
| Political Position | **-0.15**  **[-0.24,-0.06]** | 0.05 | -0.04  [-0.12,0.05] | 0.04 | -0.07  [-0.16,0.01] | 0.04 | -0.06  [-0.15,0.03] | 0.04 |
| Age | 0  [0,0.01] | 0 | 0  [-0.01,0.01] | 0 | 0  [-0.01,0] | 0 | 0  [-0.01,0.01] | 0 |
| LGBTQ+ Attitudes  x Context | **-0.23**  **[-0.35,-0.1]** | 0.06 | -0.11  [-0.23,0.01] | 0.06 | **-0.19**  **[-0.3,-0.07]** | 0.06 | **-0.13**  **[-0.25,-0.01]** | 0.06 |
| LGBTQ+ Attitudes  x GAAIS Positive | -0.09  [-0.2,0.01] | 0.05 | -0.03  [-0.13,0.07] | 0.05 | -0.02  [-0.12,0.08] | 0.05 | -0.06  [-0.15,0.04] | 0.05 |
| LGBTQ+ Attitudes  x GAAIS Negative | 0.03  [-0.07,0.13] | 0.05 | 0.08  [-0.02,0.18] | 0.05 | 0.07  [-0.03,0.17] | 0.05 | **0.1**  **[0,0.2]** | 0.05 |
| Context  x GAAIS Positive | -0.01  [-0.14,0.12] | 0.07 | -0.08  [-0.21,0.04] | 0.06 | -0.08  [-0.2,0.05] | 0.06 | 0.01  [-0.11,0.13] | 0.06 |
| Context  x GAAIS Negative | -0.02  [-0.15,0.11] | 0.07 | 0.03  [-0.1,0.15] | 0.06 | 0.05  [-0.07,0.17] | 0.06 | 0.03  [-0.09,0.15] | 0.06 |
| LGBTQ+ Attitudes  x Context  x GAAIS Positive | 0.02  [-0.12,0.16] | 0.07 | 0.05  [-0.09,0.18] | 0.07 | -0.02  [-0.15,0.11] | 0.07 | 0.07  [-0.06,0.2] | 0.07 |
| LGBTQ+ Attitudes  x Context  x GAAIS Negative | 0.04  [-0.12,0.19] | 0.08 | 0.05  [-0.1,0.2] | 0.08 | 0.02  [-0.13,0.17] | 0.08 | 0.02  [-0.13,0.17] | 0.08 |
| **(b)**  **Environmental Concerns (exploratory)** | Willingness to Act | | Trust | | Procedural Fairness | | Distributive Fairness | |
|  | Mean  [95% *HDI*] | *SD* | Mean  [95% *HDI*] | *SD* | Mean  [95% *HDI*] | *SD* | Mean  [95% *HDI*] | *SD* |
| Intercept | 0.08  [-0.29,0.52] | 0.2 | 0.26  [-0.07,0.69] | 0.19 | 0.31  [-0.06,0.79] | 0.22 | 0.13  [-0.22,0.6] | 0.21 |
| Environmentalist Attitudes | **0.14**  **[0.05,0.24]** | 0.05 | **0.1**  **[0.01,0.18]** | 0.04 | **0.17**  **[0.08,0.26]** | 0.05 | **0.17**  **[0.08,0.27]** | 0.05 |
| Context | **-0.52**  **[-0.65,-0.38]** | 0.07 | **-0.19**  **[-0.31,-0.07]** | 0.06 | **-0.35**  **[-0.48,-0.23]** | 0.06 | **-0.28**  **[-0.41,-0.15]** | 0.07 |
| GAAIS Positive | 0.05  [-0.04,0.14] | 0.04 | **0.28**  **[0.2,0.36]** | 0.04 | **0.19**  **[0.11,0.28]** | 0.04 | **0.26**  **[0.18,0.35]** | 0.04 |
| GAAIS Negative | 0.02  [-0.07,0.11] | 0.04 | **0.15**  **[0.07,0.23]** | 0.04 | 0.08  [0,0.16] | 0.04 | **0.11**  **[0.02,0.19]** | 0.04 |
| Political Position | **-0.15**  **[-0.24,-0.06]** | 0.05 | -0.04  [-0.12,0.03] | 0.04 | -0.05  [-0.14,0.03] | 0.04 | 0  [-0.09,0.08] | 0.04 |
| Age | 0  [0,0.01] | 0 | 0  [-0.01,0] | 0 | 0  [-0.01,0] | 0 | 0  [0,0.01] | 0 |
| Env. Attitudes  x Context | **-0.28**  **[-0.4,-0.16]** | 0.06 | -0.02  [-0.13,0.09] | 0.06 | **-0.14**  **[-0.26,-0.02]** | 0.06 | -0.1  [-0.22,0.02] | 0.06 |
| Env. Attitudes  x GAAIS Positive | -0.04  [-0.12,0.05] | 0.04 | -0.07  [-0.14,0.01] | 0.04 | -0.06  [-0.14,0.03] | 0.04 | -0.04  [-0.12,0.04] | 0.04 |
| Env. Attitudes  x GAAIS Negative | 0.05  [-0.05,0.15] | 0.05 | 0.04  [-0.04,0.13] | 0.04 | 0.08  [-0.01,0.17] | 0.05 | 0.02  [-0.07,0.11] | 0.05 |
| Context  x GAAIS Positive | 0.01  [-0.12,0.14] | 0.07 | -0.07  [-0.18,0.05] | 0.06 | -0.03  [-0.16,0.09] | 0.06 | 0  [-0.12,0.12] | 0.06 |
| Context  x GAAIS Negative | 0.05  [-0.08,0.18] | 0.07 | 0.03  [-0.09,0.14] | 0.06 | -0.02  [-0.14,0.09] | 0.06 | 0  [-0.13,0.12] | 0.06 |
| Env. Attitudes  x Context  x GAAIS Positive | 0.09  [-0.05,0.23] | 0.07 | 0.03  [-0.09,0.16] | 0.06 | 0.08  [-0.04,0.21] | 0.07 | 0.08  [-0.05,0.21] | 0.07 |
| Env. Attitudes  x Context  x GAAIS Negative | 0  [-0.15,0.15] | 0.08 | 0.02  [-0.11,0.15] | 0.07 | -0.06  [-0.2,0.08] | 0.07 | -0.02  [-0.16,0.13] | 0.07 |
| **(c)**  **Political Position (exploratory)** | Willingness to Act | | Trust | | Procedural Fairness | | Distributive Fairness | |
|  | Mean  [95% *HDI*] | *SD* | Mean  [95% *HDI*] | *SD* | Mean  [95% *HDI*] | *SD* | Mean  [95% *HDI*] | *SD* |
| Intercept | 0.17  [-0.31,0.67] | 0.25 | 0.22  [-0.15,0.67] | 0.21 | 0.38  [-0.02,0.85] | 0.22 | 0.16  [-0.23,0.63] | 0.22 |
| Political Position | **-0.2**  **[-0.29,-0.12]** | 0.04 | -0.05  [-0.13,0.02] | 0.04 | **-0.13**  **[-0.21,-0.05]** | 0.04 | -0.06  [-0.14,0.02] | 0.04 |
| Context | **-0.44**  **[-0.53,-0.35]** | 0.05 | **-0.19**  **[-0.29,-0.09]** | 0.05 | **-0.34**  **[-0.43,-0.24]** | 0.05 | **-0.24**  **[-0.32,-0.15]** | 0.04 |
| GAAIS Positive | 0.06  [-0.02,0.14] | 0.04 | **0.33**  **[0.26,0.4]** | 0.04 | **0.2**  **[0.13,0.28]** | 0.04 | **0.28**  **[0.2,0.35]** | 0.04 |
| GAAIS Negative | 0.03  [-0.05,0.1] | 0.04 | **0.12**  **[0.05,0.19]** | 0.04 | **0.09**  **[0.01,0.16]** | 0.04 | **0.11**  **[0.04,0.19]** | 0.04 |
| Age | 0  [0,0.01] | 0 | 0  [0,0.01] | 0 | 0  [-0.01,0] | 0 | 0  [0,0.01] | 0 |
| Political Position  x Context | **0.18**  **[0.09,0.27]** | 0.04 | 0.09  [0,0.18] | 0.05 | **0.21**  **[0.12,0.3]** | 0.05 | **0.11**  **[0.03,0.19]** | 0.04 |
| Political Position  x GAAIS Positive | 0.03  [-0.05,0.11] | 0.04 | **0.08**  **[0,0.16]** | 0.04 | **0.11**  **[0.03,0.19]** | 0.04 | 0.07  [-0.01,0.15] | 0.04 |
| Political Position  x GAAIS Negative | -0.02  [-0.11,0.07] | 0.05 | -0.07  [-0.15,0.01] | 0.04 | -0.05  [-0.13,0.03] | 0.04 | -0.07  [-0.16,0.01] | 0.04 |
| Context  x GAAIS Positive | 0.01  [-0.08,0.1] | 0.05 | -0.06  [-0.16,0.04] | 0.05 | -0.02  [-0.12,0.07] | 0.05 | 0.04  [-0.04,0.13] | 0.04 |
| Context  x GAAIS Negative | 0.03  [-0.06,0.12] | 0.05 | 0.01  [-0.08,0.11] | 0.05 | -0.01  [-0.1,0.09] | 0.05 | -0.02  [-0.1,0.06] | 0.04 |
| Political Position  x Context  x GAAIS Positive | -0.02  [-0.11,0.07] | 0.05 | -0.05  [-0.15,0.06] | 0.05 | -0.04  [-0.13,0.06] | 0.05 | -0.05  [-0.13,0.04] | 0.04 |
| Political Position  x Context  x GAAIS Negative | -0.08  [-0.18,0.02] | 0.05 | -0.04  [-0.15,0.06] | 0.05 | -0.07  [-0.17,0.03] | 0.05 | 0  [-0.09,0.08] | 0.05 |
| *Note*. Model converged successfully with split $\hat{R}$ = 1 for all estimated parameters. Context is a binary variable with liberal/left-wing direction as the reference level. GAAIS Negative values are reverse-coded. Bold emphasises 0 ∉ 95% *HDI*. | | | | | | | | |

| Table E5: Model Comparison Statistics Using Expected Log Pointwise Predictive Density (ELPD_LOO_), Leave-One-Out Information Criterion (LOO-IC), and Bayes Factor (2logBF) Between Pre-Registered and Exploratory Models in Experiment 1, 2, & 3. | | | | | |
| --- | --- | --- | --- | --- | --- |
|  |  | LOO-IC (SE) | ELPD_LOO_ (SE) | ELPD_diff_ (SE) | 2logBF |
| E1 | | | | | |
|  | Pre-reg’d model | 3094.75 (72.99) | -1547.38 (36.49) | 0 (0) | 0 |
|  | Exploratory model | 3113.32 (73.24) | -1556.66 (36.62) | -9.28 (4.31) | -71.33 |
| E2 | | | | | |
|  | Pre-reg’d model | 2375.97 (60.69) | -1187.99 (30.34) | 0 (0) | 0 |
|  | Exploratory model | 2401.09 (59.63) | -1200.54 (29.82) | -12.56 (3.79) | -77.62 |
| E3a: LGBTQ+ rights | | | | | |
|  | Pre-reg’d model | 2979.78 (73.51) | -1489.89 (36.75) | 0 (0) | 0 |
|  | Exploratory model | 3000.67 (74.19) | -1500.33 (37.09) | -10.44 (4.05) | -22.96 |
| E3b: Environmentalism | | | | | |
|  | Pre-reg’d model | 2897.5 (66.13) | -1448.75 (33.06) | 0 (0) | 0 |
|  | Exploratory model | 2923.25 (66.7) | -1461.63 (33.35) | -12.88 (3.43) | -33.14 |
| E3c: Political Position | | | | | |
|  | Pre-reg’d model | 5325.13 (102.22) | -2662.57 (51.11) | 0 (0) | 0 |
|  | Exploratory model | 5347.74 (102.38) | -2673.87 (51.19) | -11.3 (5.25) | -28.84 |
| *Note*. ELPD_LOO_ and LOO-IC were computed for each model individually, whereas ELPD_diff_ were obtained by comparing against the model with the lowest ELPD_LOO_ score. Bayes factors were computed for exploratory three-way interaction models against the denominator pre-registered full models. | | | | | |

| Table E6: Bayesian *r*^2^ for Each Outcome Variable of Pre-Registered and Exploratory Models in Experiment 1, 2, & 3. | | | | | |
| --- | --- | --- | --- | --- | --- |
|  |  | Pre-reg’d model | | Exploratory model | |
|  |  | Mean [95% *HDI*] | *SD* | Mean [95% *HDI*] | *SD* |
| E1 | | | | | |
|  | Willingness to Act | 0.18 [0.12, 0.27] | 0.04 | 0.19 [0.12, 0.27] | 0.04 |
|  | Trust | 0.18 [0.09, 0.27] | 0.05 | 0.21 [0.12, 0.3] | 0.05 |
|  | Perceived Fairness | 0.24 [0.15, 0.32] | 0.04 | 0.27 [0.18, 0.35] | 0.04 |
| E2 | | | | | |
|  | Willingness to Act | 0.13 [0.07, 0.19] | 0.03 | 0.15 [0.09, 0.21] | 0.03 |
|  | Trust | 0.04 [0.01, 0.08] | 0.02 | 0.06 [0.03, 0.1] | 0.02 |
|  | Perceived Fairness | 0.05 [0.02, 0.09] | 0.02 | 0.08 [0.04, 0.13] | 0.02 |
| E3a: LGBTQ+ rights | | | | | |
|  | Willingness to Act | 0.16 [0.11, 0.21] | 0.03 | 0.17 [0.12, 0.22] | 0.03 |
|  | Trust | 0.13 [0.09, 0.17] | 0.02 | 0.15 [0.11, 0.21] | 0.03 |
|  | Procedural Fairness | 0.11 [0.07, 0.15] | 0.02 | 0.13 [0.08, 0.17] | 0.02 |
|  | Distributive Fairness | 0.13 [0.09, 0.18] | 0.02 | 0.17 [0.11, 0.22] | 0.03 |
| E3b: Environmental Concerns | | | | | |
|  | Willingness to Act | 0.12 [0.08, 0.16] | 0.02 | 0.13 [0.09, 0.17] | 0.02 |
|  | Trust | 0.18 [0.13, 0.24] | 0.03 | 0.21 [0.15, 0.27] | 0.03 |
|  | Procedural Fairness | 0.13 [0.09, 0.18] | 0.02 | 0.16 [0.11, 0.21] | 0.03 |
|  | Distributive Fairness | 0.15 [0.11, 0.21] | 0.03 | 0.18 [0.12, 0.23] | 0.03 |
| E3c: Political Position | | | | | |
|  | Willingness to Act | 0.45 [0.39, 0.5] | 0.03 | 0.46 [0.4, 0.51] | 0.03 |
|  | Trust | 0.23 [0.15, 0.32] | 0.04 | 0.26 [0.18, 0.35] | 0.04 |
|  | Procedural Fairness | 0.31 [0.23, 0.38] | 0.04 | 0.33 [0.26, 0.4] | 0.04 |
|  | Distributive Fairness | 0.51 [0.44, 0.57] | 0.03 | 0.52 [0.45, 0.58] | 0.03 |
|  | | | | | |

# Appendix F: Posterior Predictive checks

## F.1: E1 & E2: Pre-registered & exploratory models


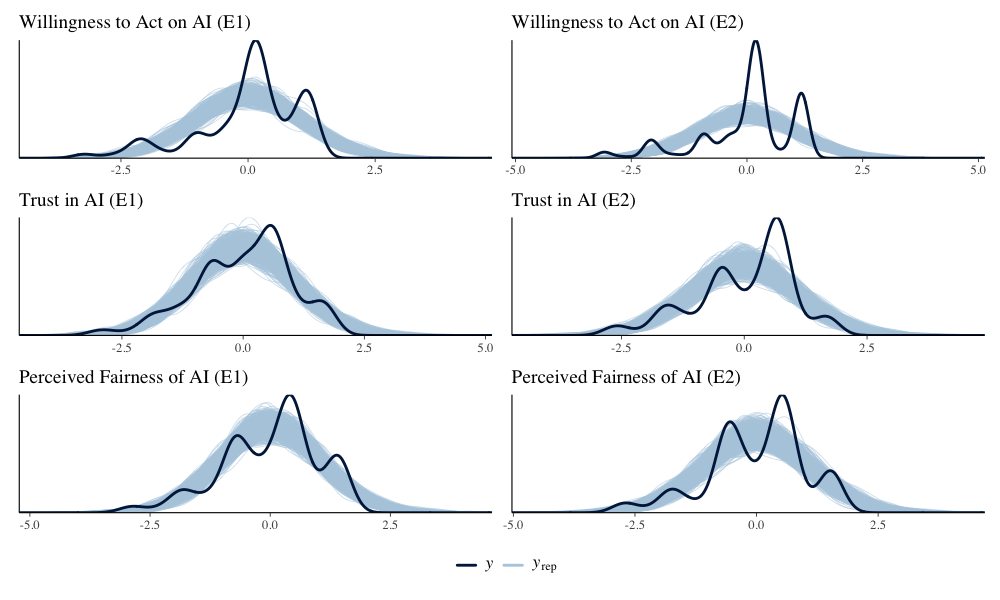


Figure F1: Comparison between distributions of observed data and model estimates for Willingness to Act on AI verdicts, Trust in AI, Fairness Perception of AI, replicated with 1000 samples from the posterior predictive distribution of E1 & E2 full models.


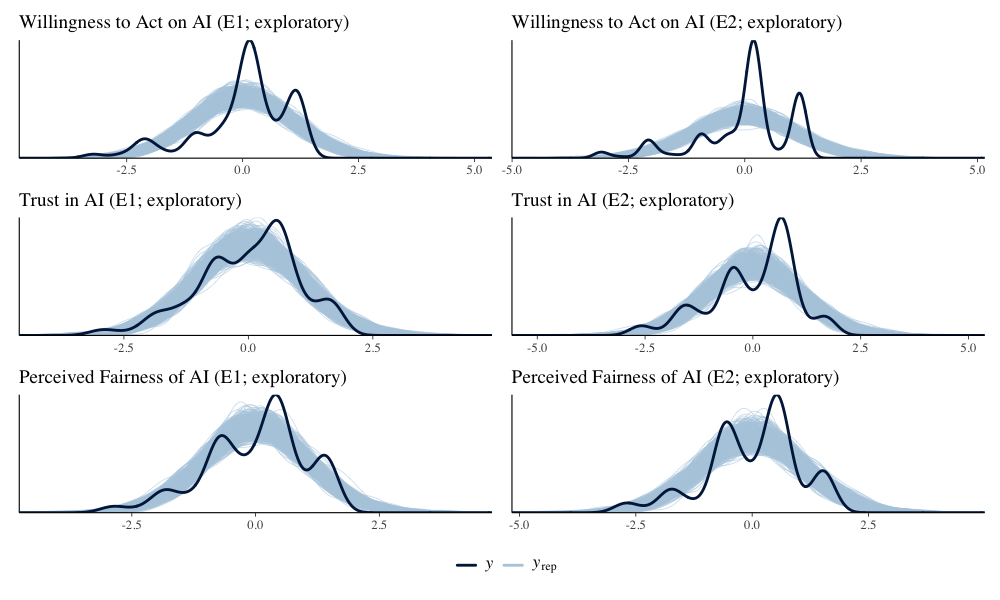


Figure F2: Comparison between distributions of observed data and model estimates for Willingness to Act on AI verdicts, Trust in AI, Fairness Perception of AI, replicated with 1000 samples from the posterior predictive distribution of E1 & E2 exploratory three-way interaction model.

## F.2: E3: Pre-registered & exploratory models


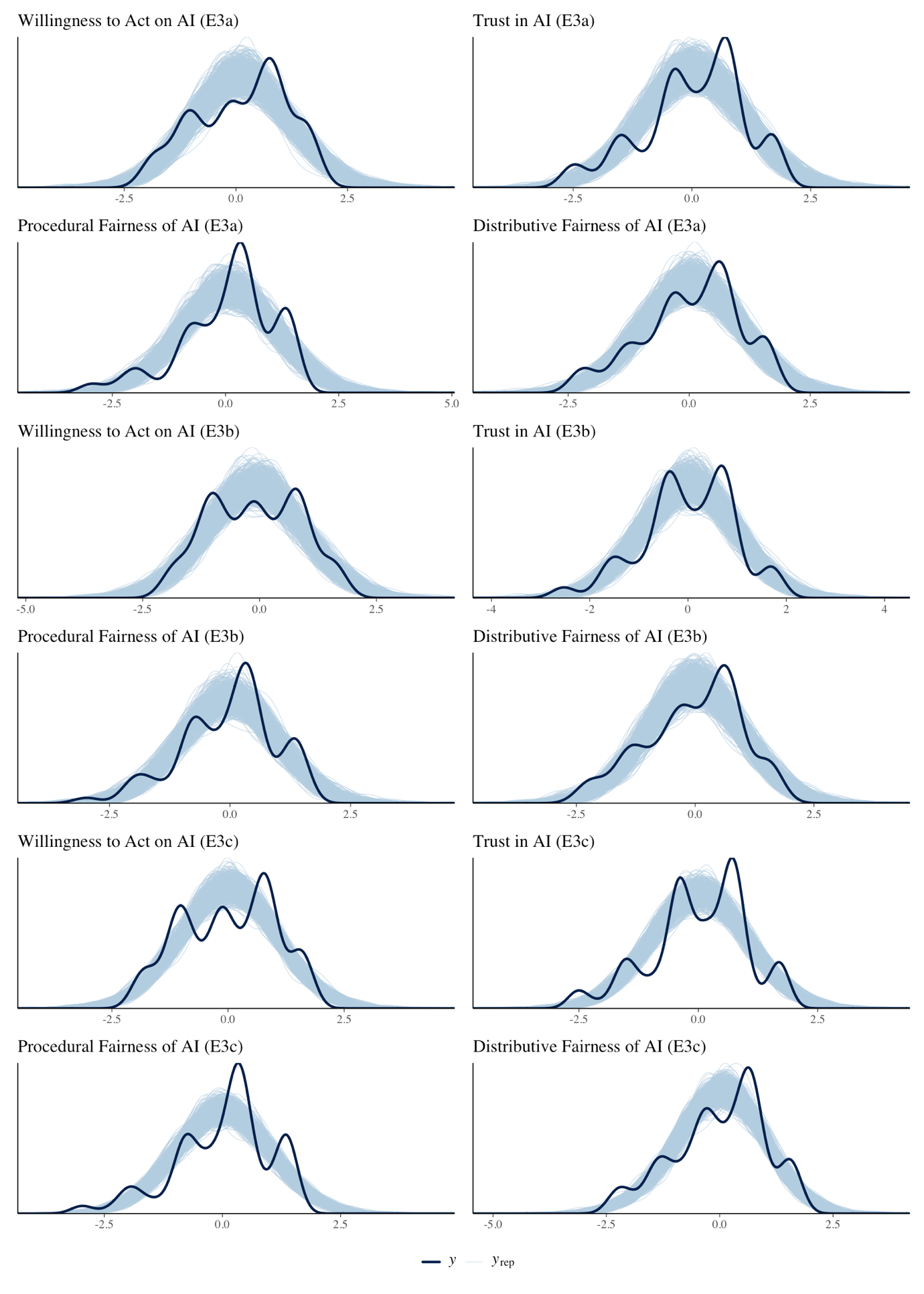


Figure F3: Comparison between distributions of observed data and model estimates for Willingness to Act on AI verdicts, Trust in AI, perception of Procedural Fairness and Distributive Fairness of AI, replicated with 1000 samples from the posterior predictive distribution of E3 model for LGBTQ+ Rights (E3a), Environmentalism (E3b), and general political position (E3c).


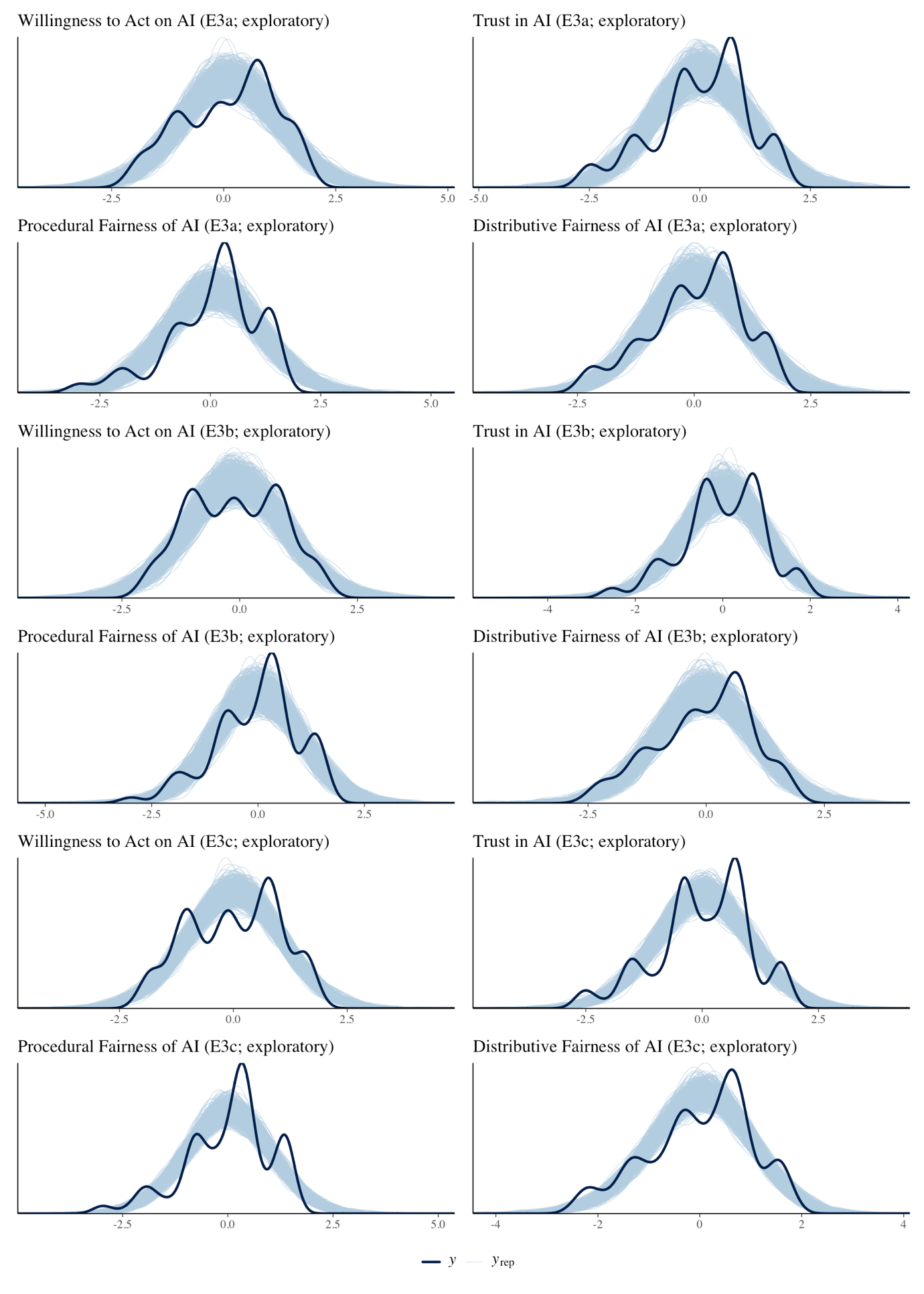


Figure F4: Comparison between distributions of observed data and model estimates for Willingness to Act on AI verdicts, Trust in AI, perception of Procedural Fairness and Distributive Fairness of AI, replicated with 1000 samples from the posterior predictive distribution of E3 exploratory three-way interaction model for LGBTQ+ Rights (E3a), Environmentalism (E3b), and general political position (E3c).

1. Schepman, A., & Rodway, P. (2020). Initial validation of the general attitudes towards artificial intelligence scale. *Computers in Human Behavior Reports*, *1*, 100014. <https://doi.org/10.1016/j.chbr.2020.100014> [↑](#footnote-ref-1)
2. Schepman, A., & Rodway, P. (2022). The general attitudes towards artificial intelligence scale (GAAIS): Confirmatory validation and associations with personality, corporate distrust, and general trust. *International Journal of Human–Computer Interaction*, 1-18. <https://doi.org/10.1080/10447318.2022.2085400> [↑](#footnote-ref-2)
